# Polymorphic Molecular Signatures in Variable Regions of the *Plasmodium falciparum var2csa* DBL3x Domain Are Associated with Virulence in Placental Malaria

**DOI:** 10.3390/pathogens11050520

**Published:** 2022-04-28

**Authors:** Eldin Talundzic, Stephen Scott, Simon O. Owino, David S. Campo, Naomi W. Lucchi, Venkatachalam Udhayakumar, Julie M. Moore, David S. Peterson

**Affiliations:** 1 Malaria Branch, Division of Parasitic Diseases and Malaria, Center for Global Health, Centers for Disease Control and Prevention, Atlanta, GA 30329, USA; wrj5@cdc.gov (E.T.); frd9@cdc.gov (N.W.L.); vxu0@cdc.gov (V.U.); 2 Department of Infectious Diseases, University of Georgia, Athens, GA 30602, USA; sscott4@augusta.edu; 3 Boehringer Ingelheim Animal Health, Athens, GA 30601, USA; sowinoo@gmail.com; 4 Molecular Epidemiology and Bioinformatics Laboratory, Division of Viral Hepatitis, Centers for Disease Control and Prevention, Atlanta, GA 30329, USA; fyv6@cdc.gov; 5 Department of Infectious Diseases and Immunology, University of Florida, Gainesville, FL 32611, USA; 6 Center for Tropical and Emerging Global Diseases, University of Georgia, Athens, GA 30602, USA

**Keywords:** placental malaria, VAR2CSA, chondroitin sulfate A, polymorphism, low birth weight, chronic infection, virulence

## Abstract

The *Plasmodium falciparum* protein VAR2CSA allows infected erythrocytes to accumulate within the placenta, inducing pathology and poor birth outcomes. Multiple exposures to placental malaria (PM) induce partial immunity against VAR2CSA, making it a promising vaccine candidate. However, the extent to which VAR2CSA genetic diversity contributes to immune evasion and virulence remains poorly understood. The deep sequencing of the *var2csa* DBL3X domain in placental blood from forty-nine primigravid and multigravid women living in malaria-endemic western Kenya revealed numerous unique sequences within individuals in association with chronic PM but not gravidity. Additional analysis unveiled four distinct sequence types that were variably present in mixed proportions amongst the study population. An analysis of the abundance of each of these sequence types revealed that one was inversely related to infant gestational age, another was inversely related to placental parasitemia, and a third was associated with chronic PM. The categorization of women according to the type to which their dominant sequence belonged resulted in the segregation of types as a function of gravidity: two types predominated in multigravidae whereas the other two predominated in primigravidae. The univariate logistic regression analysis of sequence type dominance further revealed that gravidity, maternal age, placental parasitemia, and hemozoin burden (within maternal leukocytes), reported a lack of antimalarial drug use, and infant gestational age and birth weight influenced the odds of membership in one or more of these sequence predominance groups. Cumulatively, these results show that unique *var2csa* sequences differentially appear in women with different PM exposure histories and segregate to types independently associated with maternal factors, infection parameters, and birth outcomes. The association of some *var2csa* sequence types with indicators of pathogenesis should motivate vaccine efforts to further identify and target VAR2CSA epitopes associated with maternal morbidity and poor birth outcomes.

## 1. Introduction

Nearly 12 million cases of malaria occur in pregnant women every year, resulting in thousands of deaths and adverse birth outcomes [1,2]. First and second pregnancies are at the highest risk, particularly with *Plasmodium falciparum* infection, which contributes to more than 800,000 cases of low birth weight [2] and as many as 363,000 infant deaths annually [3].

*P. falciparum* has evolved to utilize VAR2CSA, a unique variant of the highly polymorphic, multidomain erythrocyte membrane protein-1 (PfEMP1) family, to anchor infected erythrocytes (IE) to chondroitin sulfate A (CSA) within the intervillous space of the placenta [4]. The resulting syndrome, termed placental malaria (PM), is characterized by significant placental pathology [5,6]. PM results in low birth weight [7] by means of preterm delivery [8], maternal anemia [9], and intrauterine growth restriction due to disrupted maternofetal nutrient and gas exchange [10].

Multiple exposures to malaria during pregnancy promote the development of protective, inhibitory antibodies against VAR2CSA [11]. Although primigravid women also develop anti-VAR2CSA antibodies early in gestation [12,13], their persistent susceptibility to PM suggests that either this response is inadequate or relatively virulent parasites predominate in this group.

The role of VAR2CSA in PM pathogenesis has galvanized efforts to define which regions of this multidomain protein are critical for pathogenesis and therefore relevant targets for a vaccine. Current vaccine development efforts are focusing on the N-terminal region (ID1-DBL2X-ID2a) of VAR2CSA, believed to represent the minimal CSA binding region [14,15]. This region exhibits significant polymorphism [16], which may explain why recent VA2CSA vaccine trials have failed to generate cross-reactive protective immunity [17,18]. Other domains of VAR2CSA, including DBL3X, are also potentially immunologically relevant [19,20,21,22], and, similar to more N-terminal regions, DBL3X exhibits significant polymorphism between isolates [23,24]. Nonetheless, like DBL2X, DBL3X exhibits less polymorphism relative to other domains of VAR2CSA [25,26]. This is particularly interesting given the proposed role for DBL3X in maintaining the VAR2CSA structure [27,28,29] and its potential participation in CSA binding. As a follow-up to previous work showing a high level of diversity in DBL3X [24], and to examine the extent to which specific polymorphisms are associated with pathological outcomes of PM, we investigated the genetic complexity of DBL3X in placental parasites isolated from pregnant women naturally exposed to malaria in holoendemic western Kenya.

This work reveals a high number of unique DBL3X sequences, particularly in cases of chronic PM, and divergent sequence types and motifs that segregate significantly on the basis of gravidity, high density and chronic placental parasitemia, self-reported anti-malarial drug use, and birth outcomes.

## 2. Results

### 2.1. Number of Unique Sequences per Patient Is Elevated in Association with Chronic PM but Not Gravidity

Using a traditional sequencing approach, we previously identified an average of 10 unique DBL3X sequences in placental blood from Kenyan women [24]. To expand on those observations, a next-generation sequencing approach was used to assess DBL3X diversity in parasite populations isolated from the placental blood of 49 western Kenyan women recruited at parturition. Relative to the 26 multigravidae included in the study, the 23 primigravidae were younger, had higher placental parasite burdens, tended to be more likely to have histological evidence of chronic infection (as evidenced by the presence of malarial pigment (hemozoin [30])), and delivered lower birth weight infants (Table 1).

**Figure 1:**
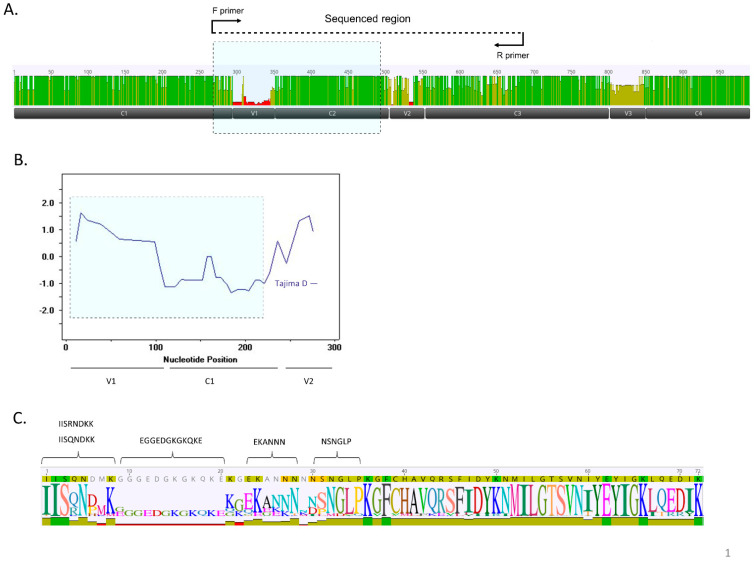
Deep sequencing of a region spanning 420 base pairs within the *var2csa* DBL3X domain reveals extensive diversity that falls into four unique groups. (**A**) Two highly variable (V1–V2) and conserved regions (C2) of the DBL3X domain were sequenced. (**B**) Tajima’s D was calculated over the first 250 nucleotides of the sequenced region using a sliding window of 10 base pairs. (**C**) The sequence logo represents a translation of the V1–C2 region, which yields a 74 amino acid peptide. regions of conserved amino acid sites and motifs of the protein sequence alignment which is comprised of conserved, variable, and indel regions (N = 522). Motifs previously reported to be associated with gravidity are indicated [24].

Next-generation sequencing provided enhanced sensitivity, yielding 127,721 high-quality reads (Supplementary Tables S1 and S2 and Supplementary Figure S1) from the 49 tested placental blood samples, covering two highly variable (V1–V2) and one conserved (C2) region of DBL3X (Figure 1A).

A de novo contig assembly analysis approach (described in Section 4) identified 522 contigs. For all samples, a subset of contigs accounts for a majority of the reads, suggesting that in most infections certain parasite genotypes are overrepresented relative to others present. At the individual patient level, the most common contig is referred to as the “dominant sequence”. In two separate 454 sequencing runs, DNA from the clonal line 3D7 and a patient previously found to be clonal (ID = 895) [24] were included as a control and in both cases yielded a single contig upon analysis. As shown in Supplementary Table S3, within the dataset of 522 contigs, 35 sequence pairs share 99% or greater identity, representing identical or near-identical sequences present in different study subjects. This includes 11 pairs sampled from geographically distinct study sites (Siaya vs. Kisumu, Kenya) at different times during the six-year sampling period. Therefore, accounting for identical sequences shared between different samples, 487 contigs were unique, yielding 127 unique sequences at the amino acid level. Seventy-six of the seventy-nine unique sequences observed in our previous work [24] were also found in this study. Rarefaction analysis revealed an accumulation curve that did not reach an asymptote (Supplementary Figure S2), suggesting that additional sampling may identify additional variants.

**Figure 2:**
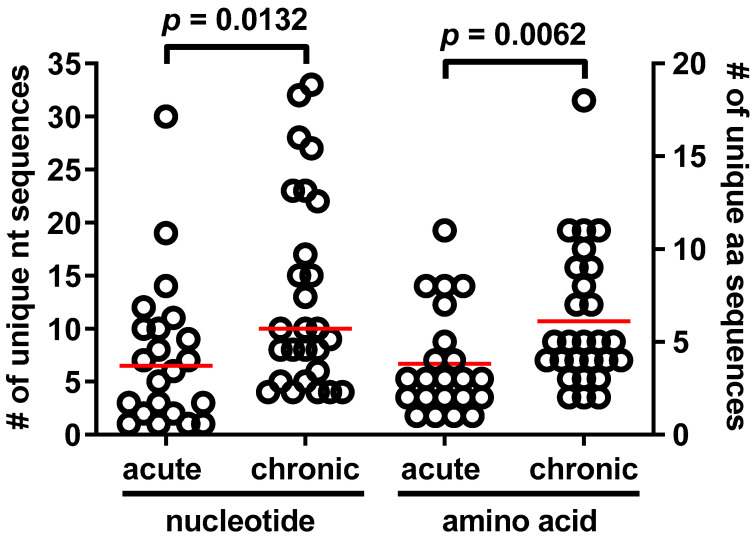
Number of unique *var2csa* sequences is positively influenced by histologically confirmed chronicity of placental malaria infection.

The number of unique nucleotide sequences within a patient sample (Tables S1 and S2) was independent of gravidity (primigravid: median = 6; 95% confidence interval (CI), 4, 22; range 1–33; multigravid: median = 9.5; CI, 5, 13.3; range 1–30, *p* = 0.3997). The same was true for peptide sequences (primigravid: median = 4; CI, 2, 7; range 1–18; multigravid: median = 4; CI, 3, 8; range 1–11, *p* = 0.5246). However, unique DNA and peptide sequence numbers were higher with chronic PM as defined by histopathological parameters (Figure 2). Univariate linear regression modeling did not reveal the associations of peptide sequence numbers with other laboratory or clinical parameters (see Methods; data not shown).

Patients were stratified according to the histological categorization of infection chronicity (Section 4) and the number of unique sequences calculated. Following the translation of sequences to amino acids, unique amino acid sequences were also ascertained. Groups were compared by the Mann–Whitney U test. Red line represents the median.

### 2.2. Association of Short Peptide Motifs in DBL3X with Gravidity and PM Outcome Parameters

Within the first 150 base pairs of the sequenced region (encompassing V1; see Figure 1A) Tajima’s D index approached 3, indicating that balancing selection, possibly immune-driven, is very high in this subdomain region (Figure 1B). Given previous findings of associations between gravidity and specific amino acid motifs in this region of DBL3X [24,31], the V1–C2 region (Figure 1A) was similarly examined. Five motifs were observed (Figure 1C; Table 2). Seventy-eight percent of the sequences contained either an IISQNDKK motif, which tended to be slightly more prevalent in primigravidae, or a less frequently observed IISRNPMK motif, which was more highly represented in multigravidae (Table 2). EGGEDGKGKQKE was observed in less than a fifth of the sequences but was more prevalent in multigravidae. The EKANNN motif predominated in primigravidae (Table 2). The highly represented NSNGLP motif was equivalently distributed independent of gravidity.

Regression analysis was used to assess associations between the carriage of these motifs and clinical and laboratory parameters, treated as dependent variables (Table S4). This analysis approach facilitates the identification of associations between individual motifs and patient parameters while controlling for the presence of other motifs. Among continuous variables, the length of gestation (gestational age at birth) was significantly associated with the EGGEDGKGKQKE motif: for each single increase in sequence number bearing this motif, the gestational age increased by 0.289 weeks (SEM: 0.119; *p* = 0.0238). For the motif EKANNN, each increase in sequence number with this motif resulted in a 0.28% increase in leukocytes bearing hemozoin in the placenta (*p* = 0.0238; Table S4). Among categorical variables, no statistically significant associations were observed for this motif (Table S4). The IISQNDKK, IISRNPMK, and NSNGLP motifs showed no significant associations with any of the tested parameters. 

We used the recently published data reporting the high-resolution structure of full-length VAR2CSA [28] to determine the location of the region of the DBL3x domain containing the motifs. As shown in Figure S3, the motifs (green region) lie in a loop that falls between major helical structures of the DBL3x domain (in blue). Based on the structure, it appears that this loop is on the external face of the DBL3x domain and not buried within it. This may account for the ability of this region to accommodate the indel found in this area.

### 2.3. Sequence Analysis Identifies Four Types That Associate with Gravidity and Infection Parameters

**Figure 3:**
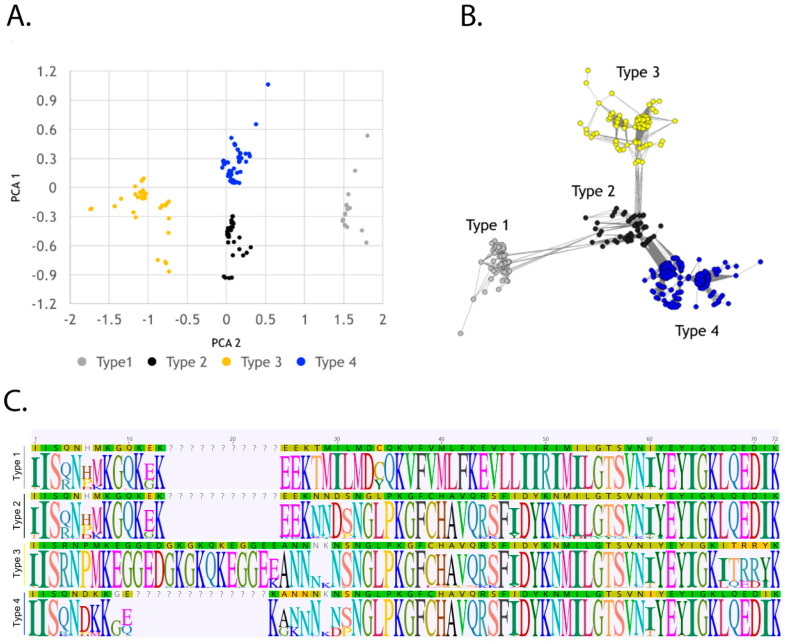
Polymorphic DBL3X sequences cluster into four discreet types. Using the genetic distance matrix from the amino acid alignment of the 522 contigs, (**A**) Ordinal Multidimensional Scaling (OMDS) and (**B**) the minimum spanning tree network using the k-step method reveal the distinct clustering of sequences into four types. (**C**) Alignments of sequences identified in the OMDS and minimum spanning network analysis were used to construct sequence logo representations of the four major sequence types identified.

Because the variability observed in DBL3X spans the bulk of the V1 region (Figure 1), an analytical approach that considered the full-length sequences (as opposed to short peptides) was next undertaken. Specifically, ordinal multidimensional scaling (OMDS) and k-step network analysis were used to visualize the genetic distance matrix obtained from the alignment of sequences unique at the amino acid level. We chose to use multidimensional scaling and k-step network analysis as complementary approaches to address the question of the current level of separation between the sequences. Both methods revealed distinct clustering into four sequence types (Figure 1D and Figure 3A,B). The detection of these types was not geographically or temporally restricted; they were consistently observed in patients recruited across multiple years and in two sites in western Kenya (Kisumu, 2002–2004) and Siaya (2004–2008; Supplementary Figure S4) that are ~70 km apart by road.

While the number of sequence types within a patient sample did not vary as a function of gravidity (primigravid: median = 2; CI, 1, 3; range 1–3; multigravid: median = 2; CI, 2, 3; range 1–3, *p* = 0.9867), in keeping with elevated sequence and peptide sequence number with chronic PM, the same relationship was evident for sequence types (acute: median = 2; CI, 1, 2; range 1–3; chronic: median = 2; CI, 2, 3; range 1–3, *p* = 0.0476). To test associations between the four identified sequence types and disease outcomes, regression analysis with clinical and laboratory parameters treated as dependent variables was performed, with the 127 unique sequences considered at the amino acid level and stratified by type (Table 3). As above, this analysis approach allows the identification of associations between individual sequence types and patient parameters while controlling for the presence of other types in the same patient. For each single increase in the carriage of a unique type 1 sequence, gestational age decreased by 0.646 weeks (SEM: 0.209; *p* = 0.0036; Table 3). Type 3 sequence number was negatively associated with percent placental parasitemia (coefficient ± SEM: −0.363 ± 0.163, *p* = 0.0311; Table 3). Among categorical variables, each single increase in carriage of a unique type 4 sequence yielded 3.26-fold increased odds for a histological assessment of chronic infection (CI: 1.41, 7.52; *p* = 0.0056; Table 3). Type 3 sequence carriage was associated with significantly reduced odds of having a percentage of placental parasitemia in the upper quartile (OR (CI): 0.184 (0.042, 0.805), *p* = 0.0245; Table 3).

### 2.4. Association of Infection Outcomes with Sequence Type Dominance 

**Figure 4:**
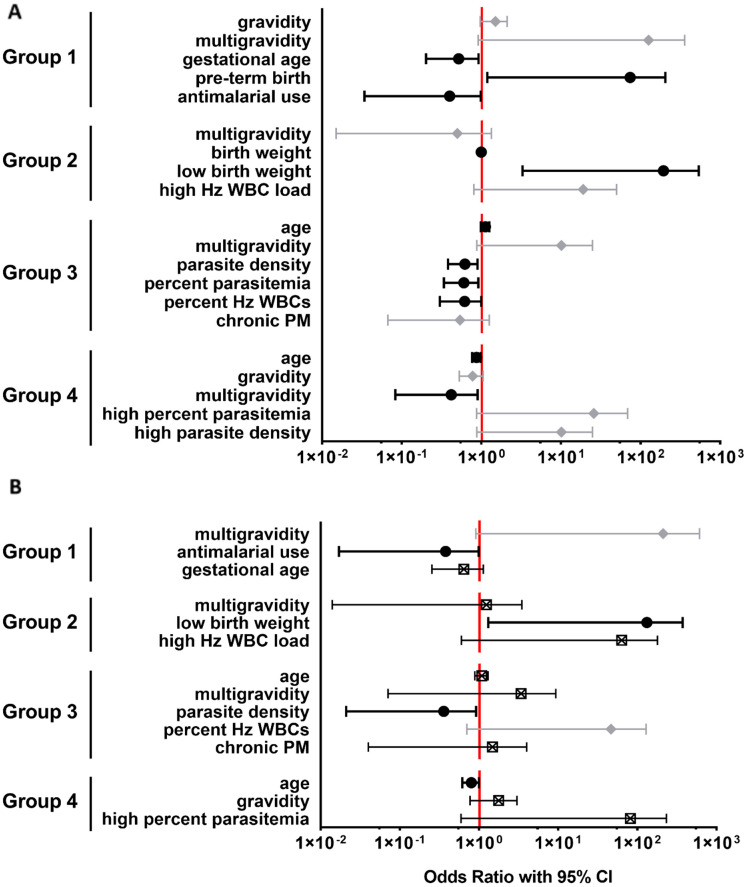
Univariate and multivariate logistic regression modeling reveals the association of DBL3X sequence type dominance with clinically relevant maternal and infant outcome variables.Forest plots show the results of univariate (**A**) and multiple (**B**) logistic regression analysis of dominant type carriers versus non-carriers for each type, defined as groups (all analysis results are provided in Tables S5 and S6). (**A**) Odds ratios (with 95% confidence interval) for statistically significant results are shown with round symbols in black; results with 0.5 > *p* > 0.1 are shown in gray with diamond symbols. (**B**) Multiple logistic regression models considered both significant and trending (0.5 > *p* > 0.1) variables. Odds ratios (with 95% confidence interval) for statistically significant results are shown with round symbols in black; results with 0.5 > *p* > 0.1 are shown in gray with diamond symbols, and results with *p* > 0.1 are shown with crossed squares in black. Variable names and descriptions are summarized in Table 1 and Table 3 and Section 4.

The assessment of unique sequences per patient revealed that, typically, one unique sequence predominates in each patient (Supplementary Figure S4). To assess which clinical parameters influence sequence dominance, “groups” were defined according to the type to which the most prevalent unique sequence within each individual patient belongs (i.e., the predominance of a type 1 sequence triggered assignment to group 1). Interestingly, 7/7 women in group 1 and 8/10 in group 3 were multigravid, whereas 5/6 in group 2 and 16/26 in group 4 were primigravid. Univariate logistic regression revealed that gravidity, maternal age, placental parasitemia, placental hemozoin within leukocytes, reported lack of antimalarial drug use, infant gestational age, and infant birth weight influence the odds of membership in one or more of these sequence predominance groups (Figure 4A). Other clinical and laboratory parameters were not associated with sequence dominance (Table S5). Logistic regression modeling revealed that for each one-week increase in gestational age, the odds of group 1 membership decreased by more than 50% (*p* = 0.0300), and indeed, were 15.6 times more likely to undergo preterm birth (*p* = 0.0365; Figure 4A, Table S5). Group 1 members also were 83% less likely than non-members to self-report the use of anti-malarial drugs (*p* = 0.0473; Figure 4A, Table S5). Multivariate modeling adjusting for gestational age and being a multigravida confirmed the risk for no antimalarial drug use in this group (*p* = 0.0491). Group 2 members had more than 22 times greater odds (*p* = 0.0319) of having a low-birth-weight baby relative to nonmembers (Figure 4A, Table S5). Multivariate modeling controlling for all of these factors revealed a high persistent risk for low birth weight in group 2 members (*p* = 0.0319; Figure 4B, Table S6). For every log increase in placental parasite density (*p* = 0.0136), percentage of placental parasitemia (*p* = 0.0211), and percentage of placental leukocytes bearing hemozoin (*p* = 0.0490), there was >40% decreased risk for membership in group 3 (Figure 4A, Table S5). The inclusion of placental parasite density in a multivariate model adjusting for age, being a multigravida, placental hemozoin burden, and chronic PM revealed that only it remained significant, with each log increase in placental parasite load yielding 86% reduced odds of being a group 3 member (Figure 4B, Table S6). Finally, each one-year increase in age yielded a 13% decreased odds (*p* = 0.0245) for group 4 membership, a group also strongly associated with primigravidity (*p* = 0.0325; Figure 4A, Table S5). In a model adjusting for age, gravidity, and being in the upper quartile percentage of placental parasitemia, younger age emerged as the defining attribute for group 4 membership (*p* = 0.0486; Figure 4B, Table S6).

## 3. Discussion

Var genes are highly polymorphic [32], facilitating immune evasion in *P. falciparum* [33]. Thus, var members that are associated with virulence are arguably optimal antigenic targets for vaccine development [34]. Despite relative conservation in comparison to other members of the var gene family [35], *var2csa* varies considerably among laboratory *P. falciparum* isolates [36] and globally disparate patient samples [16,25,31,37]. Nonetheless, VAR2CSA is increasingly subject to antibody recognition over successive malaria-exposed pregnancies [11,15,38] and has therefore emerged as a vaccine candidate for protecting women against PM [36]. Indeed, early work found that human antibody recognition of VAR2CSA was strain-transcendent, leading to predictions of broad cross-protection by these responses [39,40]. However, more recent work has challenged this notion [41,42], raising concerns about the impact of polymorphisms on the efficacy of a vaccine targeting VAR2CSA [16], which were indeed borne out in a recent VAR2CSA vaccine trial [18]. There is considerable interest in the N-terminal region (DBL1X, DBL2X, CIDR) of VAR2CSA, which is necessary and sufficient for CSA binding [19,42], and is the target for naturally acquired antibodies capable of interfering with cytoadhesion [40]. Our previous conventional DNA sequencing of DBL3X revealed a high degree of diversity [24]. Considerable polymorphism is also evident in the ID1-DBL2Xb segment [25,42]. Because VAR2CSA polymorphism may retard the acquisition of cross-protective immune recognition [43] by altering linear or conformational immune epitopes proximally and distally to mutated residues, it is critical to elucidate the genetic diversity of this protein and associated impacts on function [44], capacity to promote pathogenesis, and host immune recognition. Two VAR2CSA-based vaccines, PAMVAC and PRIMVAC, were recently tested in clinical trials [17,18]. Both vaccines are comprised of the N-terminal domains shown to be the minimal regions required for binding to CSA. They differ in that one is based on the VAR2CSA sequence from the 3D7 parasite strain, the other on the FCR3 strain. Neither vaccine elicits a broadly reactive immune response capable of recognizing the diverse VA2CSA sequences present in field populations of the parasite [17,18,25]. This may be related to the absence of conformational epitopes that are only present in the full-length protein [45,46]. Additional regions of the protein may contribute to binding to CSA, and are indispensable to protein structure, as demonstrated recently with the cryo-electron microscopic analysis of full-length VAR2CSA [28,29]. Another recent study utilizing small angle X-ray scattering and the single particle reconstruction of negative-stained electron micrographs suggested that a secondary site for CSA binding may utilize the domain’s C-terminal to the minimal CSA binding domain, including DBL3X [26].

This study used targeted deep sequencing, which affords the sensitive detection of parasite diversity, including minor variants, to probe the diversity of *var2csa* in placental parasites obtained from women naturally exposed to endemic *P. falciparum* (Table 1).

Most of the DBL3X sequences reported here are novel, and rarefaction analysis suggests that additional sequencing may identify further variants (Figure S2). Here, we analyzed a DBL3X region spanning 420bp in length, identifying 487 unique sequences (based on the nucleotide level). This is comparable to another study that probed diversity in a 54bp region in DBL3X and identified 222 unique sequences [20]. Because the sequenced region represents <5% of the total *var2csa* coding region, the actual number of unique *var2csa* sequences is likely to be far greater in this population. Benaventa et al. have examined the diversity of full-length *var2csa* sequences from geographically diverse parasites and, consistent with our work, found a significant degree of diversity in DBL3x, though lower nucleotide diversity than domains within the N-terminal minimal binding region. While that study reported a lower percentage of indel positions in DBL3x relative to the DBL2x domain, our work demonstrates that motifs within this indel loop region are associated with clinically relevant parameters in PM, suggesting that the same may be true of indel regions in other domains. It will be interesting to see if these indels also map to putative surface-exposed loop regions, as we demonstrated for the DBL3x domain (Figure S3). Although independent of gravidity, the number of unique DBL3X sequences (Figure 2) and the number of sequence types on a per-patient basis were significantly higher in women with histologically-confirmed chronic PM. In this area of high endemicity [47], chronic PM likely represents more than one independent exposure to infection. Given the high level of diversity observed in DBL3X in this study, it would be expected that multiple exposures would yield greater within-host diversity relative to “new”, shorter-lived acute infections.

Multigravid women avoid severe outcomes of PM through the development of inhibitory antibodies that can block CSA binding [11]; opsonizing antibodies are also a significant aspect of this immune response [21]. The extent to which antibody-mediated immune pressure leads to the selection of specific *var2csa* sequence types, however, remains unknown. Consistent with our previously published work [24] and that of others [31], the data reported here show clear evidence for gravidity-associated selection in DBL3X at the sequence level, both in terms of short peptide motifs (Table 2) and the region encompassing variable subdomain V1 that shows evidence of balancing selection (Figure 3). A striking finding of the present work is the association of these peptide motifs with clinically relevant parameters in PM, infant gestational age, and placental hemozoin burden. Such observations provide critical knowledge about protein regions (and therefore epitopes) that may be essential targets of protective immunity.

Ordinal multidimensional scaling (OMDS) and k-step network analysis of the region with the highest evidence of balancing selection revealed clustering into four sequence types (Figure 3), facilitating a broader assessment of associations between *var2csa* diversity and critical indicators of PM pathogenesis. Histologically confirmed chronic PM was associated with the carriage of a higher number of these unique sequence types relative to acute infection, suggesting that in this environment, superinfection is a contributor to chronic infection. At the sequence type level, a type 1 sequence number was related to reduced infant gestational age at birth (Table 3). The multivariate analysis of sequence type dominance confirmed that type 1 sequence carriage is associated with preterm birth (Figure 4). While a number of multigravidae in this study did not fall into this sequence dominance group, it is interesting that all members of this group were multigravidae, suggesting a tendency for these parasites to establish infection only in this group. Interestingly, type 1 dominance also predicts self-reported failure to use anti-malarial drugs and is the only factor that maintained significance in a model controlling for the other factors. Thus, type 1 sequence-bearing parasites may not be particularly competitive relative to others but can establish a low-level infection in multigravidae who avoid the use of antimalarial drugs and are immune to other, potentially “primigravida-tropic”, VAR2CSA types, such as type 2. The recent evidence of significant variability in antigenicity and CSA binding affinity amongst VAR2CSA variants may be related to the presence of these sequence types in DBL3X or similar sequence types in other domains of this protein [44]. While a type 2 sequence number did not associate significantly with any of the tested parameters, 83% of members in group 2 (type 2 sequence dominance) were primigravidae. Importantly, membership in this group is associated with having a low-birth-weight infant, an outcome of PM that is most prevalent in lower-order births [48]. Types 3 and 4 sequence number carriage also appeared to segregate to different PM outcomes: whereas type 3 sequence numbers were inversely related to parasitemia, increasing type 4 sequences were associated with chronic PM (Table 3). Furthermore, type 3 sequence dominance was associated with less intense placental infection (Figure 4), and this dominance group was 80% multigravidae. Group 4, in whom type 4 sequences were dominant, was 62% primigravid, ref. [20], and a younger age remained a significant factor in association with this group in multivariate analysis. Considered all together, these results suggest that the identified sequence types fall into two broad categories loosely defined by gravidity/age, infection intensity, PM chronicity, and birth outcomes. This evidence suggests that polymorphism in this region of DBL3X, or variant sequences with which these types are in linkage disequilibrium, is a key determinant for the establishment and persistence of infection in pregnant women and therefore represents a critical target for vaccine-induced immunity. Moreover, the results show clear links with critical birth outcomes (low birth weight and pre-term birth), highlighting the need to consider the importance of anti-disease and anti-pathogenesis parameters when choosing vaccine antigens. Given the striking association of type 2 sequences with a critically important outcome of PM in vulnerable primigravid women and type 4 sequences with age, targeting parasite variants of these putative virulent types in a pregnancy-specific malaria vaccine might be particularly beneficial. Our results are concordant with other work that looked at sequences of the VAR2CSA ID1-DBL2x region and found associations of specific VAR2CSA clades with low birth weight [37]. This clearly demonstrates the need for further studies that use full-length VAR2CSA sequences to investigate additional associations with pathology and birth outcome.

The association of type 1 sequences with a self-reported lack of use of anti-malarial drugs is intriguing. *P. falciparum* in this region of Kenya is known to carry multiple resistance mutations to sulfadoxine-pyrimethamine, the prophylactic drug of choice during the recruitment period for this study [49]. Similar to the situation in neighboring Tanzania [50], sulfadoxine-pyrimethamine failure is associated with a risk for elevated placental parasitemia in this population (Matthias et al. manuscript in preparation), but the determination of how drug use might influence *var2csa* diversity will require further study.

In summary, we report here exceptionally high levels of inter- and intra-patient *var2csa* DBL3X diversity. Further, we identify unique sequence types that, similar to previous reports, are influenced by gravidity [24,31] and associated with elevated placental parasitemia [20]. Our novel observations extend such associations to maternal age, anti-malarial drug use, PM chronicity, and birth outcomes, including infant birth weight and gestational age. Given the high degree of interest in developing a VAR2CSA-based vaccine to protect reproductive-age women in endemic areas [43,51], and the dependence of strain-transcendent immunity to VAR2CSA on discontinuous or conformational epitopes that may only be found in the native, full-length protein [46], we propose that continued efforts to identify virulence types through intensive sequencing efforts and association studies, such as those reported here, is essential for the identification of optimal antigens for an efficacious vaccine. 

## 4. Methods

### 4.1. Ethics Statement

The study was approved by the Kenya Medical Research Institute Ethical Review Committee and the Institutional Review Boards of the University of Georgia, the Centers for Disease Control and Prevention, and the National Institutes of Health. All participants provided informed, written consent under the auspices of these approved protocols. All methods were carried out in accordance with the approved guidelines. All data associated with this study are now anonymized.

### 4.2. Study Population

Samples and clinical data analyzed here are derived from a cross-sectional study of immune responses to malaria during pregnancy in western Kenya. Recruitment was conducted from November 2002 to September 2008 at New Nyanza Provincial General Hospital in Kisumu and Siaya District Hospital. A previous subgroup analysis of these populations revealed a higher parasite burden among the latter [52]. As a whole, this study population included a total of 1046 parturient women, ranging from gravida 1 to 11.

For the present work, a total of 60 maternal placental blood samples were selected from available placental blood thick smear malaria-positive samples. This initial set included 29 primigravid, 3 secundigravid, and 28 multigravid samples (ranging from gravida 3 to 9). An additional 26 submicroscopic but PCR-confirmed PM+ samples [30] (9 primigravid, 7 secundigravid, and 10 multigravid samples, ranging from gravida 3 to 8) were also selected. Other than PM status and gravidity, no other clinical or laboratory parameters were considered at this stage of sample selection. Aligning with a desire to compare primigravid samples to multigravidae, the secundigravid samples were restricted to technical optimization for pyrosequencing but were ultimately omitted from further consideration. Among the remaining 57 smear-positive and 19 submicroscopic samples, 19 and 9, respectively, were omitted at various stages of the study due to the consumption of all available DNA during optimization, the failure of amplification in initial PCR screens with DBL3X primers, or the failure of the sample during 454 sequencing library preparation. Ultimately, 49 placental blood samples, 10 recruited from Kisumu and 39 from Siaya, representing 23 primigravidae and 26 multigravidae, and spanning the entirety of the parent study (Figure S5), were fully analyzed. The multigravid samples represent 13 gravida 3, 8 gravida 4, 2 gravida 5, and one each of gravida 6, 8 and 9. Clinical and demographic data, collected post-partum by interview and the assessment of patient records, are summarized in Table 1.

Immediately post-expulsion, placentas were collected and maternal placental blood was obtained either by the prick method [53] or perfusion [54] under aseptic conditions. Placental parasite density was estimated by counting the number of infected erythrocytes on a Giemsa-stained thick smear of maternal placental prick blood per at least 300 white blood cells. The percentage of these white blood cells containing phagocytosed hemozoin was also recorded, and is reported as “percent hemozoin WBCs”. Because complete blood count data (using a COULTER^®^ A^c^·T™ 5diff CP; Cap Pierce) was not available for all women, the calculation of parasite density per microliter of blood used the median placental white blood cell count (12,000/uL for those HIV seronegative and 13,450/uL for those HIV seropositive) for all PM+ women in the parent study for whom complete blood count was ascertained. Percent parasitemia was assessed on Giemsa-stained thin smears of prick blood samples. A thin smear was not available for one multigravida. For two other multigravidae with a low level of infection by thick smear, the percent parasitemia was too low to enumerate accurately; these samples are therefore assigned a parasitemia of 0%. As noted in Table 1, 10 samples (3 from primigravidae and 7 from multigravidae) were blood smear-negative but PCR-positive in placental blood [30]. Gestational age was determined by the modified Dubowitz test [55], and birth weight was measured to an accuracy of 50 g within twelve hours after delivery. Peripheral hemoglobin was measured by Coulter counter, as above; data for sixteen women were not available. Relevant clinical information, including the usage of anti-malarial drugs during pregnancy, was collected from hospital records and by interview within 24 h after delivery.

Placental tissue sections were preserved and histologically assessed as described [30]. At least two discrete full-thickness regions of the placental disk were assessed. Chronic infection was defined as fibrin hemozoin score ≥2 and leukocyte hemozoin score ≥1. Acutely infected placentas scored 1 ± 1 (mean ± SD) for hemozoin-bearing inflammatory cells and 1 ± 1 for hemozoin embedded in fibrin. Chronically infected placentas scored 2 ± 1 for inflammatory cells and 3 ± 1 for fibrin. Histological sections were not available for six participants

Analytical cut-offs for high placental percent parasitemia (≥3.4%), high placental parasite density (≥23,187 parasites/uL), and high percent placental white blood cells bearing hemozoin (10.7%) were based on the upper quartile for all microscopically infected women in the parent study population.

### 4.3. Amplification and Sequencing of var2csa DBL3X Domain

The variable region of the DBL3X domain of *var2csa* was amplified from extracted DNA using previously described primers [24]. The primers were modified for 454 sequencing by including a multiplex identifier (MID), linker, and tag sequence. Each patient sample was amplified in triplicate using the modified primers. Amplicons were pooled and sequenced using a 454 GS Junior at the Georgia Genomics Facility at The University of Georgia. Figure S6 presents a flow chart overview of the next-gen sequencing pipeline.

### 4.4. Data Quality Filtering

A total of 158,573 sequence reads were obtained, and low-quality reads were removed on the basis of a defined cut-off (i.e., length and quality scores). The length cut-off was determined based on all published DBL3X sequences available as of 31 September 2013, and sequences below 320 bp or above 420 bp were omitted. Additionally, sequences with a Q20 quality score of less than 80 were discarded, leaving 127,721 sequences. All quality filtering and data analysis were performed using Geneious v8.1 [56].

Next, all sequences were BLAST searched for *P. falciparum* and non-hits were discarded. The remaining sequences, numbering 120,625, were separated by unique MIDs (multiplex identifiers) from the pooled data into patient-specific sequences. For each patient data set, sequences were trimmed of MIDs, tags, and primers prior to subsequent analysis.

### 4.5. Unique Sequence Types Analysis

For each patient sample, a mean of 3254 (SD = 1648) reads was obtained. A de novo contig assembly approach was used to identify unique sequence types using Geneious v8.1 Geneious Assembler [56] and the Velvet Short Read Assembler plugin. Each method yielded the same number of unique contigs. For each patient, contigs (i.e., unique sequence types) were obtained through the computation of overlaps between reads, the removal of false overlaps, and the construction of multiple sequence alignments. A minimum/maximum overlap of 320 bp/420 bp and identity of 98% were selected to account for the expected read length and the intrinsic 454 sequencing error of 2%. A total of 522 unique contigs were produced. Between 5 and 5880 (median = 343) reads per patient per contig were obtained. Contigs with fewer than 5 reads were omitted, corresponding to 522 unique contigs. To validate this approach in determining unique sequence types, a 3D7 clonal lineage, as well as a patient found to be clonal (ID = 895) previously [24], was included as a control in each of the 454 runs. These samples consistently yielded a single contig (e.g., one unique sequence type). All sequences have been submitted to GenBank as a PopSet with accession numbers KP275019–KP275392 and KP275406–KP275551.

Ordinal multidimensional scaling (OMDS) and k-step network analysis were used to visualize the genetic distance matrix obtained from the amino acid alignment of unique sequences. Ordinal Multidimensional Scaling (OMDS) finds the optimal display of cases in a dataset for a chosen number of dimensions and was performed with IBM SPSS (New York, NY, USA) statistics for windows. The k-step network displays all possible minimum spanning trees [57] and was built using Python 2.7. Diversity and rarefaction curves were determined using EstimateS v9.1.0 [58]. Tajima D was calculated using DnaSP v5 [59]. Additional information about the data and unique sequence types is provided in Supplementary Figure S5.

### 4.6. Motif Analysis

Sequence motif analysis was guided using two criteria: 1. The region that showed the highest evidence of balancing selection as indicated by Tajima D >1 (Figure 1B). Using the sequence region and motifs previously identified to produce DBL3X antibodies [31].

### 4.7. VAR2CSA Structure Modeling

The protein structure of VAR2CSA (Protein Data Bank: 7JGH) [28] was used as a template for structure modeling. Structure visualization and figure generation were performed using UCSF Chimera molecular analysis program [60].

### 4.8. Statistical Analysis and Graph Generation

Descriptive analysis and graphing of clinical data were performed with GraphPad Prism, v9.2.0. Non-normal data were log-transformed toward normality for statistical analysis. Comparisons of two groups with normally distributed data were conducted with an unpaired two-tailed *t*-test. Proportions were assessed by Fisher’s exact or x^2^ tests with Pearson’s correction as appropriate. Regression analyses were performed using SAS, v9.4. Continuous clinical and laboratory parameters considered were age, gravidity, placental parasite density, percent placental parasitemia, percent placental leukocytes bearing hemozoin, peripheral hemoglobin, infant birth weight, and gestational age at birth. Categorical variables were gravidity group (primigravid, multigravida), HIV serostatus, high (upper quartile)/low (lower three quartiles including submicroscopic) placental parasite density and percent placental parasitemia, placental histology group (acute/chronic), self-reported antimalarial drug use, birth weight (low (≤2500 g)/normal), and gestational age (preterm (<37 weeks)/term). To assess the extent to which these parameters influence the number of unique sequences per patient, univariate linear regression analysis was performed with continuous clinical and laboratory parameters as the dependent variable, and logistic regression was performed with categorical clinical and laboratory parameters as dependent variables. For the examination of motif and sequence type carriage, which must account for the contribution of various combinations of these features in each individual, linear regression was performed with continuous clinical and laboratory parameters as dependent variables and motif or sequence type counts as predictors; likewise, logistic regression used categorical clinical and laboratory parameters as dependent variables. For the analysis of sequence dominance, groups were defined and subjects assigned based on the most frequent sequence type found at the patient level. Analysis of this parameter assigned group membership as the dependent variable in univariate and multivariate regression analyses, with independent variables including the above described clinical and laboratory parameters. All multivariate linear and logistic regression models incorporated independent variables with *p* < 0.1 from univariate analysis.

## Figures and Tables

**Table 1:** Summary data for the study participants.

	Primigravid (n = 23)	Multigravid (n = 26)	*p* Value ^a^
Gravidity (mean, range)	1	4, 3–4.3	<0.0001
Age (years)	18 (17, 19)	25 (23, 28.3)	<0.0001
Luo ethnicity (%)	22/23 (96)	24/26 (92)	1
Resident of Siaya District (%)	16/23 (70)	23/26 (88)	0.16
Submicroscopic placental infection (%) ^b^	3/23 (13)	7/26 (27)	0.30
% Placental parasitemia ^b^	1.8 (0.5, 7.0)	0.5 (0.3, 1.2)	0.037
Placental parasite density/µL ^b^	15,502 (4723, 50, 521)	1564 (651, 4410)	0.0014
Chronic placental malaria (%) ^c^	16/20 (80)	11/23 (48)	0.056
% Hemozoin-bearing WBC ^d^	4.3 (1.2, 8.4)	1.0 (0, 4.3)	0.041
HIV seropositive (%)	1/23 (4)	6/26 (23)	0.10
Peripheral hemoglobin level (g/dL) ^e^	11.6 ± 2.0	10.5 ± 2.1	0.13
Self-reported antimalarial use (%); SP use (%) ^f^	15/23 (65); 15/23 (65)	16/26 (62); 12/26 (46)	1/0.25
Infant birth weight (g)	3009 ± 383	3304 ± 436	0.015
Gestational age (wks) ^g^	38.3 ± 1.0	38.2 ± 1.4	0.61
Male birth (%)	10/23 (43)	16/26 (62)	0.26

^a^ Statistical tests: proportions compared using a two-tailed Fisher’s exact test; hemoglobin level and birth weight are compared by unpaired *t*-test Welch’s and shown as mean ± SEM; others by Mann–Whitney U test, shown as median (interquartile range (IQR). ^b^ Parasitemia data represent values for clients with blood smear detectable infection; a thin smear was not available for one thick smear-positive multigravid woman. Submicroscopic infection is defined as smear-negative and PCR-positive (see Methods). ^c^ Placenta sections for the histological categorization of infection were not available for three primigravid and three multigravid women; chronic infection is defined as in Methods. ^d^ White blood cells (WBC) bearing hemozoin relative to total WBCs counted on a thick placental blood smear. ^e^ Data were not available for six primigravid and nine multigravid women. ^f^ Based on self-reporting; one primigravid woman reported antimalarial drug use but could not name the type of drug taken; one multigravid woman reported using an unspecified antimalarial other than sulfadoxine/pyrimethamine, metakelphin, or quinine. None reported use of chloroquine. ^g^ Data were not available for two primigravid women.

**Table 2:** Distribution of previously identified amino acid motifs in DBL3X.

Motif	No. Observed	% of Total Population (n = 522)	Primigravid n (%)	Multigravid n (%)	*p*
IISQNDKK	273	51	144 (27)	129 (24)	0.0571
IISRNPMK	138	26	52 (9)	86 (16)	0.0008
EGGEDGKGKQKE	94	18	37 (7)	60 (11)	0.0313
EKANNN	222	41	125 (24)	97 (18)	0.0049
NSNGLP	328	62	159 (29)	169 (32)	0.4237

**Table 3:** Regression analysis of factors associated with DBL3X sequence type carriage.

**Continuous Variables**												
	**Type 1**			**Type 2**			**Type 3**			**Type 4**		
	**Co-efficient**	**SEM**	***p***	**Co-efficient**	**SEM**	***p***	**Co-efficient**	**SEM**	***p***	**Co-efficient**	**SEM**	***p***
Age	0.737	0.973	0.453	−0.877	0.586	0.142	0.764	0.616	0.222	−0.806	0.414	0.0580
Gravidity	0.371	0.325	0.260	−0.362	0.196	0.0711	0.306	0.206	0.145	−0.170	0.138	0.225
Placental parasite density ^a^	−0.036	0.298	0.905	0.190	0.179	0.296	−0.347	0.189	0.0727	0.173	0.127	0.179
Percent placental parasitemia ^a^	−0.0340	0.258	0.896	0.169	0.154	0.281	**−0.363**	**0.163**	**0.0311**	0.207	0.109	0.0648
Placental hemozoin burden ^b^	−0.147	0.266	0.584	0.155	0.123	0.215	−0.177	0.129	0.176	0.127	0.0907	0.170
Peripheral hemoglobin ^c^	−0.080	0.471	0.866	−0.125	0.257	0.631	−0.407	0.264	0.133	0.075	0.177	0.675
Infant birth weight	45.4	77.8	0.563	−64.0	46.8	0.179	60.9	49.3	0.223	−31.2	33.1	0.350
Gestational age at birth	**−0.646**	**0.209**	**0.0036**	−0.0676	0.127	0.596	0.0913	0.132	0.494	−0.0219	0.0899	0.809
**Categorical Variables**												
	**Type 1**			**Type 2**			**Type 3**			**Type 4**		
	**OR (95% CI)**		***p***	**OR (95% CI)**		***p***	**OR (95% CI)**		***p***	**OR (95% CI)**		***p***
Gravidity group	3.85 (0.763, 19.4)		0.103	0.621 (0.342, 1.13)		0.118	1.55 (0.872, 2.74)		0.135	0.835 (0.594, 1.17)		0.298
Upper quartile parasite density	0.231 (0.020, 2.72)		0.245	1.14 (0.698, 1.85)		0.611	0.657 (0.333, 1.30)		0.226	1.12 (0.777, 1.60)		0.552
Upper quartile percent parasitemia	0.454 (0.064, 3.20)		0.428	1.78 (0.863, 3.68)		0.118	**0.184 (0.042, 0.805)**		**0.0245**	1.19 (0.696, 2.04)		0.524
Upper quartile hemozoin-WBCs	0.684 (0.100, 4.66)		0.698	1.52 (0.852, 2.69)		0.158	1.47 (0.799, 2.71)		0.215	1.02 (.0641, 1.61)		0.947
Placental histology group ^d^	0.305 (0.073, 1.28)		0.105	4.12 (0.910, 18.6)		0.0661	0.615 (0.305, 1.24)		0.176	**3.26 (1.41, 7.52)**		**0.0056**
Anemia	0.521 (0.135, 2.01)		0.345	1.14 (0.628, 2.05)		0.674	1.43 (0.830, 2.46)		0.198	1.28 (0.892, 1.85)		0.179
HIV serostatus	0.994 (0.416, 2.37)		0.990	0.315 (0.047, 2.12)		0.236	1.11 (0.601, 2.04)		0.742	1.27 (0.852, 1.91)		0.239
Self-reported antimalarial drug use ^e^	0.896 (0.427, 1.88)		0.773	1.57 (0.682, 3.63)		0.288	1.14 (0.642, 2.02)		0.656	0.863 (0.604, 1.23)		0.418
Low birth weight	0.956 (0.258, 3.55)		0.947	1.53 (0.881, 2.65)		0.132	0.907 (0.415, 1.98)		0.806	1.20 (0.754, 1.92)		0.437
Preterm birth	2.66 (0.891, 7.96)		0.0797	0.884 (0.161, 4.85)		0.887	0.604 (0.114, 3.19)		0.552	0.832 (0.287, 2.41)		0.735

Univariate regression analyses with continuous (linear regression) and categorical (logistic regression) variables as dependent variables and numbers of unique (amino acid level) sequences within each type at the patient level as predictors. Statistically significant results (*p* < 0.05) are bolded. Among categorical variables, the gravidity group tests multigravid status, parasitemia and hemozoin analyses test presence in the upper quartile, histology group tests chronic PM, anemia tests hemoglobin <11 g/dL, HIV tests HIV seropositivity, self-reported antimalarial drug use tests the reported use of drugs, low birth weight tests birth weight ≤2500 g, and preterm birth tests gestational age <37 weeks. ^a^ determined as summarized in Table 1 and Methods; parasitemia analyses performed using log-transformed data. Percentage of placental parasitemia analysis omits one multigravida for whom a placental thin smear was unavailable. ^b^ percent of white blood cells on a thick smear bearing phagocytosed hemozoin; log-transformed data. ^c^ data are missing for 16 clients. ^d^ analysis omits six patients for whom histological analysis was unavailable. ^e^ reported use of antipyretics is categorized as no antimalarial drug use.

## Data Availability

The data presented in this study are available in the main text, figures, and tables.

## Supplementary Materials

The following supporting information can be downloaded at: https://www.mdpi.com/article/10.3390/pathogens11050520/s1, Table S1: Summary of data obtained for primigravid women; Table S2: Summary of data obtained for multigravid women; Table S3: Sequence pairs sharing 99% or higher identity; Table S4: Univariate regression analysis of factors associated with amino acid motif carriage in DBL3X; Table S5: Univariate logistic regression analysis of sequence type dominance; Table S6: Multiple logistic regression analysis of sequence type dominance. Figure S1: Summary of total number of quality reads used. Shown on the x-axis is the total number of sequences and y-axis the sequence lengths in base pairs (bp); Figure S2: Rarefaction curve of *var2csa* unique sequence types for entire study population (N = 49). The computed refraction curve (in solid green) shows the expected average rate of unique sequence types that would be produced as a result of repeated deep sequencing. Upper and lower 95% confidence intervals (CI) for species richness are shown by dashed and dotted red lines, respectively; Figure S3: VAR2CSA and DBL3X motif region. A ribbon representation of VAR2CSA (grey), DBL3X domain (blue). Dotted circles and atom stick representation show the CSA binding site (orange) and specific motif region (green) associated with gravidity and placental malaria outcomes; Figure S4: DBL3X sequence types are consistently found independently of time and location of patient recruitment. The graph depicts proportional distribution of sequence types by year of patient recruitment. The tale at right summarizes number of patients recruited each year (stratified by gravidity group), number of unique sequences contributed by those patients, and the mean number of unique sequences per patient; Figure S5: Unique DBL3X sequences are not proportionally represented at the individual patient level. The graphs depict the number of unique contigs within each patient. Notably, most patients have a single dominant contig. Patient 895 is depicted twice, representing two separate deep sequencing runs. This patient was previously identified (REF) as having a single unique sequence. DNA from the clonal laboratory isolate, 3D7, was also included as a control, and shows, in two separate runs, a single contig. Similar colors across individual patients do *not* imply sequence similarity; Figure S6: Schematic overview of the overall sequencing process.

## References

[1] Dellicour S., Tatem A.J., Guerra C.A., Snow R.W., ter Kuile F.O. (2010). Quantifying the number of pregnancies at risk of malaria in 2007: A demographic study. PLoS Med..

[2] WHO (2021). World Malaria Report 2021.

[3] WHO (2020). World Malaria Report 2020.

[4] Goel S., Gowda D.C. (2011). How specific is Plasmodium falciparum adherence to chondroitin 4-sulfate?. Trends Parasitol..

[5] Brabin B.J., Romagosa C., Abdelgalil S., Menéndez C., Verhoeff F.H., McGready R., Fletcher K.A., Owens S., d’Alessandro U., Nosten F. (2004). The sick placenta-the role of malaria. Placenta.

[6] Nosten F., Rogerson S.J., Beeson J.G., McGready R., Mutabingwa T. (2004). and Brabin, B. Malaria in pregnancy and the endemicity spectrum: What can we learn?. Trends Parasitol..

[7] Brabin B.J. (1983). An analysis of malaria in pregnancy in Africa. Bull. World Health Organ..

[8] Sullivan A.D., Nyirenda T., Cullinan T., Taylor T., Harlow S.D., James S.A., Meshnick S.R. (1999). Malaria infection during pregnancy: Intrauterine growth retardation and preterm delivery in Malawi. J. Infect. Dis..

[9] Gilles H.M., Lawson J.B., Sibelas M., Voller A., Allan N. (1969). Malaria, anaemia and pregnancy. Ann. Trop. Med. Parasitol..

[10] Brabin B. (1991). An assessment of low birthweight risk in primiparae as an indicator of malaria control in pregnancy. Int. J. Epidemiol..

[11] Fried M., Nosten F., Brockman A., Brabin B.J., Duffy P.E. (1998). Maternal antibodies block malaria. Nature.

[12] O’Neil-Dunne I., Achur R.N., Agbor-Enoh S.T., Valiyaveettil M., Naik R.S., Ockenhouse C.F., Zhou A., Megnekou R., Leke R., Taylor D.W. (2001). Gravidity-dependent production of antibodies that inhibit binding of Plasmodium falciparum-infected erythrocytes to placental chondroitin sulfate proteoglycan during pregnancy. Infect. Immun..

[13] Cox S.E., Staalsoe T., Arthur P., Bulmer J.N., Hviid L., Yeboah-Antwi K., Kirkwood B.R., Riley E.M. (2005). Rapid acquisition of isolate-specific antibodies to chondroitin sulfate A-adherent plasmodium falciparum isolates in Ghanaian primigravidae. Infect. Immun..

[14] Srivastava A., Gangnard S., Dechavanne S., Amirat F., Bentley A.L., Bentley G.A., Gamain B. (2011). Var2CSA minimal CSA binding region is located within the N-terminal region. PLoS ONE.

[15] Tuikue-Ndam N., Deloron P. (2015). Developing vaccines to prevent malaria in pregnant women. Expert Opin. Biol. Ther..

[16] Doritchamou J., Sabbagh A., Jespersen J.S., Renard E., Salanti A., Nielsen M.A., Deloron P., Tuikue Ndam N. (2015). Identification of a Major Dimorphic Region in the Functionally Critical N-Terminal ID1 Domain of VAR2CSA. PLoS ONE.

[17] Mordmuller B., Sulyok M., Egger-Adam D., Resende M., de Jongh W.A., Jensen M.H., Smedegaard H.H., Ditlev S.B., Soegaard M., Poulsen L. (2019). First-in-human, Randomized, Double-blind Clinical Trial of Differentially Adjuvanted PAMVAC, A Vaccine Candidate to Prevent Pregnancy-associated Malaria. Clin. Infect. Dis..

[18] Sirima S.B., Richert L., Chêne A., Konate A.T., Campion C., Dechavanne S., Semblat J.P., Benhamouda N., Bahuaud M., Loulergue P. (2020). PRIMVAC vaccine adjuvanted with Alhydrogel or GLA-SE to prevent placental malaria: A first-in-human, randomised, double-blind, placebo-controlled study. Lancet Infect. Dis..

[19] Dahlback M., Jørgensen L.M., Nielsen M.A., Clausen T.M., Ditlev S.B., Resende M., Pinto V.V., Arnot D.E., Theander T.G., Salanti A. (2011). The chondroitin sulfate A-binding site of the VAR2CSA protein involves multiple N-terminal domains. J. Biol. Chem..

[20] Rovira-Vallbona E., Monteiro I., Bardají A., Serra-Casas E., Neafsey D.E., Quelhas D., Valim C., Alonso P., Dobaño C., Ordi J. (2013). VAR2CSA signatures of high Plasmodium falciparum parasitemia in the placenta. PLoS ONE.

[21] Lambert L.H., Bullock J.L., Cook S.T., Miura K., Garboczi D.N., Diakite M., Fairhurst R.M., Singh K., Long C.A. (2014). Antigen reversal identifies targets of opsonizing IgGs against pregnancy-associated malaria. Infect. Immun..

[22] Dechavanne S., Srivastava A., Gangnard S., Nunes-Silva S., Dechavanne C., Fievet N., Deloron P., Chêne A., Gamain B. (2015). Parity-dependent recognition of DBL1X-3X suggests an important role of the VAR2CSA high-affinity CSA-binding region in the development of the humoral response against placental malaria. Infect. Immun..

[23] Gamain B., Smith J.D., Viebig N.K., Gysin J., Scherf A. (2007). Pregnancy-associated malaria: Parasite binding, natural immunity and vaccine development. Int. J. Parasitol..

[24] Talundzic E., Shah S., Fawole O., Owino S., Moore J.M., Peterson D.S. (2012). Sequence polymorphism, segmental recombination and toggling amino acid residues within the DBL3X domain of the VAR2CSA placental malaria antigen. PLoS ONE.

[25] Benavente E.D., Oresegun D.R., de Sessions P.F., Walker E.M., Roper C., Dombrowsk J.G., de Souza R.M., Marinho C.R.F., Sutherland C.J., Hibberd M.L. (2018). Global genetic diversity of *var2csa* in Plasmodium falciparum with implications for malaria in pregnancy and vaccine development. Sci. Rep..

[26] Bewley M.C., Gautam L., Jagadeeshaprasad M.G., Gowda D.C., Flanagan J.M. (2020). Molecular architecture and domain arrangement of the placental malaria protein VAR2CSA suggests a model for carbohydrate binding. J. Biol. Chem..

[27] Gangnard S., Lewit-Bentley A., Dechavanne S., Srivastava A., Amirat F., Bentley G.A., Gamain B. (2015). Structure of the DBL3X-DBL4epsilon region of the VAR2CSA placental malaria vaccine candidate: Insight into DBL domain interactions. Sci. Rep..

[28] Ma R., Lian T., Huang R., Renn J.P., Petersen J.D., Zimmerberg J., Duffy P.E., Tolia N.H. (2021). Structural basis for placental malaria mediated by Plasmodium falciparum VAR2CSA. Nat. Microbiol..

[29] Wang K., Dagil R., Lavstsen T., Misra S.K., Spliid C.B., Wang Y., Gustavsson T., Sandoval D.R., Vidal-Calvo E.E., Choudhary S. (2021). Cryo-EM reveals the architecture of placental malaria VAR2CSA and provides molecular insight into chondroitin sulfate binding. Nat. Commun..

[30] Avery J.W., Smith G.M., Owino S.O., Sarr D., Nagy T., Mwalimu S., Matthias J., Kelly L.F., Poovassery J.S., Middii J.D. (2012). Maternal malaria induces a procoagulant and antifibrinolytic state that is embryotoxic but responsive to anticoagulant therapy. PLoS ONE.

[31] Dahlback M., Rask T.S., Andersen P.H., Nielsen M.A., Ndam N.T., Resende M., Turner L., Deloron P., Hviid L., Lund O. (2006). Epitope mapping and topographic analysis of VAR2CSA DBL3X involved in P. falciparum placental sequestration. PLoS Pathog..

[32] Su X.Z., Heatwole V.M., Wertheimer S.P., Guinet F., Herrfeldt J.A., Peterson D.S., Ravetch J.A., Wellems T.E. (1995). The large diverse gene family var encodes proteins involved in cytoadherence and antigenic variation of Plasmodium falciparum-infected erythrocytes. Cell.

[33] Roberts D.J., Craig A.G., Berendt A.R., Pinches R., Nash G., Marsh K., Newbold C.I. (1992). Rapid switching to multiple antigenic and adhesive phenotypes in malaria. Nature.

[34] Rowe J.A., Kyes S.A. (2004). The role of Plasmodium falciparum var genes in malaria in pregnancy. Mol. Microbiol..

[35] Trimnell A.R., Kraemer S.M., Mukherjee S., Phippard D.J., Janes J.H., Flamoe E., Su X.Z., Awadalla P., Smith J.D. (2006). Global genetic diversity and evolution of var genes associated with placental and severe childhood malaria. Mol. Biochem. Parasitol..

[36] Bockhorst J., Lu F., Janes J.H., Keebler J., Gamain B., Awadalla P., Su X.Z., Samudrala R., Jojic N., Smith J.D. (2007). Structural polymorphism and diversifying selection on the pregnancy malaria vaccine candidate VAR2CSA. Mol. Biochem. Parasitol..

[37] Patel J.C., Hathaway N.J., Parobek C.M., Thwai K.L., Madanitsa M., Khairallah C., Kalilani-Phiri L., Mwapasa V., Massougbodji A., Fievet N. (2017). Increased risk of low birth weight in women with placental malaria associated with P. falciparum VAR2CSA clade. Sci. Rep..

[38] Ataide R., Mayor A., Rogerson S.J. (2014). Malaria, primigravidae, and antibodies: Knowledge gained and future perspectives. Trends Parasitol..

[39] Avril M., Cartwright M.M., Hathaway M.J., Smith J.D. (2011). Induction of strain-transcendent antibodies to placental-type isolates with VAR2CSA DBL3 or DBL5 recombinant proteins. Malar. J..

[40] Avril M., Hathaway M.J., Srivastava A., Dechavanne S., Hommel M., Beeson J.G., Smit J.D., Gamain B. (2011). Antibodies to a full-length VAR2CSA immunogen are broadly strain-transcendent but do not cross-inhibit different placental-type parasite isolates. PLoS ONE.

[41] Doritchamou J., Bigey P., Nielsen M.A., Gnidehou S., Ezinmegnon S., Burgain A., Massougbodji A., Deloron P., Salanti A., Ndam N.T. (2013). Differential adhesion-inhibitory patterns of antibodies raised against two major variants of the NTS-DBL2X region of VAR2CSA. Vaccine.

[42] Bordbar B., Tuikue Ndam N., Renard E., Jafari-Guemouri S., Tavul L., Jennison C., Gnidehou S., Tahar R., Gamboa D., Bendezu J. (2014). Genetic diversity of VAR2CSA ID1-DBL2Xb in worldwide Plasmodium falciparum populations: Impact on vaccine design for placental malaria. Infect. Genet. Evol..

[43] Dahlback M., Nielsen M.A., Salanti A. (2010). Can any lessons be learned from the ambiguous glycan binding of PfEMP1 domains?. Trends Parasitol..

[44] Renn J.P., Doritchamou J.Y.A., Tentokam B.C.N., Morrison R.D., Cowles M.V., Burkhardt M., Ma R., Mahamar A., Attaher O., Diarra B.S. (2021). Allelic variants of full-length VAR2CSA, the placental malaria vaccine candidate, differ in antigenicity and receptor binding affinity. Commun. Biol..

[45] Doritchamou J.Y.A., Suurbaar J., Ndam N.T. (2021). Progress and new horizons toward a VAR2CSA-based placental malaria vaccine. Expert Rev. Vaccines.

[46] Doritchamou J.Y.A., Renn J.P., Jenkins B., Mahamar A., Dicko A., Fried M., Duffy P.E. (2022). A single full-length VAR2CSA ectodomain variant purifies broadly neutralizing antibodies against placental malaria isolates. Elife.

[47] Githeko A.K., Brandling-Bennett A.D., Beier M., Atieli F., Owaga M., Collins F.H. (1992). The reservoir of Plasmodium falciparum malaria in a holoendemic area of western Kenya. Trans. R. Soc. Trop. Med. Hyg..

[48] Desai M., ter Kuile F.O., Nosten F., McGready R., Asamoa K., Brabin B. (2007). and Newman, R.D. Epidemiology and burden of malaria in pregnancy. Lancet Infect. Dis..

[49] Iriemenam N.C., Shah M., Gatei W., van Eijk A.M., Ayisi J., Kariuki S., Eng J.V., Owino S.O., Lal A.A., Omosun Y.O. (2012). Temporal trends of sulphadoxine-pyrimethamine (SP) drug-resistance molecular markers in Plasmodium falciparum parasites from pregnant women in western Kenya. Malar. J..

[50] Harrington W.E., Mutabingwa T.K., Muehlenbachs A., Sorensen B., Bolla M.C., Fried M., Duffy P.E. (2009). Competitive facilitation of drug-resistant Plasmodium falciparum malaria parasites in pregnant women who receive preventive treatment. Proc. Natl. Acad. Sci. USA.

[51] Menendez C., Moorthy V.S., Reed Z., Bardají A., Alonso P., Brown G.V. (2011). Development of vaccines to prevent malaria in pregnant women: WHO MALVAC meeting report. Expert Rev. Vaccines.

[52] Perrault S.D., Hajek J., Zhong K., Owino S.O., Sichangi M., Smith G., Shi Y.P., Moore J.M., Kain K.C. (2009). Human immunodeficiency virus co-infection increases placental parasite density and transplacental malaria transmission in Western Kenya. Am. J. Trop. Med. Hyg..

[53] Othoro C., Moore J.M., Wannemuehler K., Nahlen B.L., Otieno J., Slutsker L., Lal A.A., Shi Y.P. (2006). Evaluation of various methods of maternal placental blood collection for immunology studies. Clin. Vaccine Immunol..

[54] Moore J.M., Nahlen B., Ofulla A.V., Caba J., Ayisi J., Oloo A., Misore A., Nahmias A.J., Lal A.A., Udhayakumar V. (1997). A simple perfusion technique for isolation of maternal intervillous blood mononuclear cells from human placentae. J. Immunol. Methods.

[55] Dubowitz L.M., Dubowitz V., Goldberg C. (1970). Clinical assessment of gestational age in the newborn infant. J. Pediatrics.

[56] Kearse M., Moir R., Wilson A., Stones-Havas S., Cheung M., Sturrock S., Buxton S., Cooper A., Markowitz S., Duran C. (2012). Geneious Basic: An integrated and extendable desktop software platform for the organization and analysis of sequence data. Bioinformatics.

[57] Campo D.S., Dimitrova Z., Yamasaki L., Skums P., Lau D.T.Y., Vaughan G., Forbi J.C., Teo C.-E., Khudyakov Y. (2014). Next-generation sequencing reveals large connected networks of intra-host HCV variants. BMC Genom..

[58] Colwell R. (2013). EstimateS: Statistical Estimation of Species Richness and Shared Species from Samples. https://www.researchgate.net/publication/247642760_Statistical_Estimation_of_Species_Richness_and_Shared_Species_from_Samples.

[59] Librado P., Rozas J. (2009). DnaSP v5: A software for comprehensive analysis of DNA polymorphism data. Bioinformatics.

[60] Pettersen E.F., Goddard T.D., Huang C.C., Couch G.S., Greenblatt D.M., Meng E.C., Ferrin T.E. (2004). UCSF Chimera--a visualization system for exploratory research and analysis. J. Comput. Chem..

