# Supplementary material for: Polymorphic Molecular Signatures in Variable Regions of the Plasmodium falciparum var2csa DBL3x Domain Are Associated with Virulence in Placental Malaria
Source: Pathogens. 2022 Apr 28;11(5):520. doi: 10.3390/pathogens11050520 (PMC9147263; doi:10.3390/pathogens11050520)
Supplement: Supplementary file 1 [file pathogens-11-00520-s001.zip › pathogens-1613503-supplementary.pdf]

Supplementary Materials

Table S1. Summary of data obtained for primigravid women.

Patient ID	Placental parasite density/uL	Percent placental parasitemia	UniqueSeq (contigs)	Copy #	Total Reads	Q Reads	Analysis Reads	Unused Q Reads	Unused Total Reads
10049	207090	45.90	2	1.01	3158	3142	3129	13	29
10079	36357	7.80	32	1.07	5113	5103	4391	712	722
10221	8233	1.00	33	1.21	3662	3616	3296	320	366
10224	90827	56.50	4	2.85	5946	5930	5884	46	62
10327	10010	2.60	23	2.13	4777	4764	4541	223	236
10340	0	0.00	1	4.14	2503	2441	2426	15	77
10398	26910	1.00	5	2.91	1975	1961	1894	67	81
10450	0	0.00	7	6.76	2298	2235	2206	29	92
10460	563	0.50	3	5.1	2443	2421	2398	23	45
10465	217	0.10	1	6.68	2330	2319	2303	16	27
10506	4333	1.30	17	4.86	4326	4318	4125	193	201
10549	520	0.40	23	1.91	1354	1173	848	325	506
10626	10877	7.10	8	2.01	2134	1472	1298	174	836
10696	58630	17.90	4	5.39	1837	1642	1419	223	418
10737	0	0.00	28	2.95	5449	5433	5206	227	243
10858	5619	0.59	4	2.3	8311	956	940	16	7371
10895	107303	6.56	1	1.1	2538	2091	2071	20	467
11202	6201	0.27	6	1.27	1037	1027	983	44	54
11318	27775	3.37	22	6.1	1249	1237	936	301	313
11320	52389	2.27	8	1.06	8137	1922	1758	164	6379
11332	2926	0.20	10	2.8	2225	588	472	116	1753
11354	44243	3.67	6	0.93	4043	1511	1403	108	2640
11434	38320	0.60	4	1	1956	1904	1844	60	112
Sum			252		78801	59206	55771	3435	23030
Mean	36967*	7.98*	11	2.93	3426	2574	2424	149	1001
SD	50329*	15.46*	10	1.92	2002	1513	1468	158	1914
Median	18894*	1.79*	6	2.30	2503	2091	2071	108	243

Total number of sequences obtained for each 454 run is shown, including sequences used for downstream analysis (e.g. = Analysis Reads). Please refer to Study population, materials and methods section for detailed information on procedures about sequence clean-up and quality assessment. *Calculation based on microscopically evident infections only.

Table S2. Summary of data obtained for multigravid women.

Gravidity	Patient ID	Placental parasite density/uL	Percent placental parasitemia	Unique Seq (contigs)	Copy #	Total Reads	Q Reads	Analysis Reads	Unused Q Reads	Unused Total Reads
4	10324	4377	0.60	19	6.68	1541	1524	773	751	768
4	10353	22187	0.80	11	2.2	5639	5625	5547	78	92
3	10380	0	0.00	7	1.02	2815	2784	2771	13	44
8	10473	0	0.00	14	5.54	2342	2306	2270	36	72
3	10486	3380	0.70	10	5.08	5445	5360	5242	118	203
3	10490	1213	0.40	13	3.18	4550	4535	4477	58	73
3	10521	0	0.00	10	7.21	2331	2322	2248	74	83
4	10551	217	0.30	10	5.98	2478	1064	873	191	1605
3	10580	0	0.00	30	9.63	4280	4037	3799	238	481
3	10608	40257	21.90	15	5.08	3419	1861	1514	347	1905
3	10612	0	0.00	3	4.23	3143	2853	2826	27	317
5	10640	0	0.00	1	9.04	3509	3488	3450	38	59
9	10694	14083	2.60	4	4.95	2039	1354	1282	72	757
4	10745	4680	2.26	9	2.45	5355	5339	5259	80	96
2	10781	1552	0.58	28	4.14	3413	3392	3139	253	274
5	10899	0	0.00	2	2.2	2256	2193	2182	11	74
4	10948	867	-	12	6.92	4280	4270	4224	46	56
4	11186	172	0.00	8	8.4	1608	1528	1477	51	131
3	11290	810	0.00	10	5.62	1291	1274	1078	196	213
3	11295	2048	1.30	8	4.55	4289	1866	1749	117	2540
3	11322	1921	0.20	9	7.41	1584	1529	1421	108	163
3	11331	646	0.10	5	1.28	2028	1982	1960	22	68
3	11335	2786	0.40	16	6.4	1555	1516	1103	413	452
4	11336	458	0.10	5	6.38	4289	1747	1541	206	2748
3	11340	1296	0.50	8	4.91	2254	2242	2061	181	193

3	11416	386	0.29	3	4.65	2393	875	588	287	1805
	Sum			270		80126	68866	64854	4012	15272
	Mean:	4959*	2.06*	10	5.19	3082	2649	2494	154	587
	SD:	10135*	5.34*	7	2.21	1294	1390	1449	160	796
	Median:	1424*	0.54*	9	5.08	2646	2217	2122	94	198

Total number of sequences obtained for each 454 run is shown, including sequences used for downstream analysis (e.g. Analysis Reads). Please refer to Study population, materials and methods section for detailed information on procedures about sequence clean-up and quality assessment. *Calculation based on microscopically evident infections only.

Table S3. Sequence pairs sharing 99% or higher identity.

Sequence 1	Sequence 2
10049.C2	10490.C7
10221.C20	10549.C14
10608.C2	11186.C2
10221.C20	10450.C6
10224.C1	10450.C4
10612.C2	11290.C10
10221.C20	10465.C1
10353.C11	10506.C11
10737.C3	10745.C5
10327.C6	10506.C4
10450.C6	10465.C1
11322.C8	11331.C4
10380.C5	10473.C5
10450.C6	10549.C14
11322.C8	11336.C2
10380.C2	11336.C1
10473.C2	10486.C3
11331.C4	11336.C2
10473.C15	10580.C1
10473.C15	10899.C1
11322.C6	11336.C3
10549.C1	10608.C2
10580.C1	11336.C1

11322.C8	11340.C6
10580.C3	10612.C2
10580.C3	11290.C10
11331.C4	11340.C6
10899.C1	11336.C1
11318.C6	11336.C1
11332.C9	11354.C4
11336.C2	11340.C6
10221.C2	10781.C4
10353.C5	10506.C5
10549.C1	11186.C2
10580.C1	10899.C1

In total, 56 sequences form 35 sequence pairs that share 99% or greater sequence identity. Sequences are named by sample number followed by contig number. These represent identical or near identical sequences found in different study subjects, on different dates and in some cases different study site locations. Sequence pairs outlined with a bold border are from different study sites, with Kisumu sequences in in the range of 10001 to 10450, and Siaya sequences represented by sample numbers > 10450.

Table S4. Univariate regression analysis of factors associated with amino acid motif carriage in DBL3X.

	IISQNDKK			IISRNPMK			EGGEDGKGKQKE			EKANNN			NSNGLP		
**Continuous variables**															
	Co-efficient	SEM	*P*	Co-efficient	SEM	*P*	Co-efficient	SEM	*P*	Co-efficient	SEM	*P*	Co-efficient	SEM	*P*
Age	-0.214	0.338	0.530	0.688	0.618	0.272	-0.228	0.608	0.710	0.0753	0.558	0.893	-0.224	0.501	0.658
Gravidity	-0.0347	0.115	0.765	0.071	0.211	0.738	0.100	0.207	0.631	-0.0628	0.190	0.743	-0.0108	0.171	0.950
Placental parasite density [a]	0.0364	0.0966	0.708	-0.0552	0.177	0.756	0.00151	0.174	0.993	0.210	0.159	0.194	-0.153	0.143	0.291
Percent placental parasitemia [a]	0.0300	0.0861	0.729	0.0694	0.157	0.661	-0.107	0.155	0.495	0.218	0.143	0.134	-0.163	0.127	0.209
Placental hemozoin burden [b]	-0.0270	0.0737	0.717	0.104	0.131	0.433	-0.0221	0.124	0.860	**0.280**	**0.119**	**0.0238**	-0.187	0.111	0.0996
Peripheral hemoglobin [c]	0.143	0.155	0.363	-0.135	0.330	0.686	-0.0456	0.279	0.871	-0.0581	0.267	0.829	0.0357	0.232	0.879
Infant birth weight	15.4	27.0	0.573	-33.3	49.4	0.504	61.7	48.6	0.211	-25.1	44.6	0.577	0.220	40.1	0.996
Gestational age at birth	*-0.141*	*0.0802*	*0.0868*	*-0.244*	*0.141*	*0.0899*	**0.289**	**0.140**	**0.0455**	0.149	0.123	0.232	0.00575	0.108	0.958

Categorical variables										
	IISQNDKK		IISRNPMK		EGGEDGKGKQKE		EKANNN		NSNGLP	
	OR (95% CI)	*P*	OR (95% CI)	*P*	OR (95% CI)	*P*	OR (95% CI)	*P*	OR (95% CI)	*P*
Gravidity group	1.02 (0.797, 1.30)	0.889	1.05 (0.672, 1.64)	0.827	1.08 (0.694, 1.70)	0.723	0.865 (0.574, 1.30)	0.489	1.04 (0.721, 1.49)	0.841
Upper quartile parasite density	1.04 (0.745, 1.46)	0.803	0.561 (0.270, 1.16)	0.120	1.07 (0.525, 2.19)	0.850	0.811 (0.440, 1.50)	0.503	1.29 (0.715, 2.32)	0.399
Upper quartile percent para-sitemia	1.13 (0.763, 1.67)	0.545	0.875 (0.449, 1.70)	0.694	0.332 (0.088, 1.26)	0.105	0.760 (0.330, 1.75)	0.521	1.32 (0.688, 2.54)	0.403
Upper quartile hemozoin-WBCs	1.07 (0.636, 1.81)	0.791	*1.99 (0.950, 4.16)*	*0.0681*	0.765 (0.382, 1.53)	0.449	1.15 (0.557, 2.38)	0.705	0.724 (0.403, 1.30)	0.278
Placental histology group [d]	1.07 (0.751, 1.53)	0.702	1.03 (0.571, 1.86)	0.922	0.848 (0.446, 1.61)	0.616	*1.80 (0.971, 3.34)*	*0.0618*	0.857 (0.494, 1.49)	0.582
Anemia	0.789 (0.564, 1.10)	0.166	1.39 (0.685, 2.82)	0.362	0.948 (0.540, 1.66)	0.852	1.29 (0.746, 2.23)	0.364	0.931 (0.568, 1.53)	0.779
Self-reported antimalarial drug use [e]	1.09 (0.815, 1.47)	0.553	0.968 (0.580, 1.61)	0.900	1.30 (0.788, 2.15)	0.305	0.972 (0.603, 1.57)	0.908	0.875 (0.579, 1.32)	0.527
HIV sero-status	0.882 (0.619, 1.26)	0.488	0.922 (0.476, 1.79)	0.809	0.943 (0.542, 1.64)	0.834	1.01 (0.567, 1.80)	0.977	1.19 (0.718, 1.98)	0.497
Low birth weight	1.11 (0.691, 1.77)	0.674	1.66 (0.801, 3.44)	0.173	*0.526 (0.248, 1.12)*	*0.0937*	0.861 (0.376, 1.97)	0.723	1.05 (0.577, 1.90)	0.882
Preterm birth	*2.43 (0.878, 6.74)*	*0.0875*	0.830 (0.117, 5.89)	0.852	1.95 (0.064, 59.4)	0.701	0.768 (0.045, 13.1)	0.855	0.367 (0.017, 8.05)	0.524

Univariate linear regression analyses with continuous (linear regression) and categorical (logistic regression) variables as dependent variable and motif numbers at the patient level as predictors. Statistically significant results ($p < 0.05$) are bolded; trending results ($0.05 < p < 0.1$) are italicized. Among categorical variables gravidity group tests multigravid status, parasitemia and hemozoin analyses test presence in the upper quartile, histology group tests chronic PM, anemia tests hemoglobin < 11 g/dL, HIV tests HIV seropositivity, self-reported antimalarial drug use tests reported use of drugs, low birth weight tests birth weight ≤2500 g, and preterm birth tests gestational age <37 weeks.

[a] determined as summarized in Table 1 and Methods; parasitemia analyses done using log-transformed data. Percent placental parasitemia analysis omits one multigravida for whom a placental thin smear was unavailable.

[b] percent of white blood cells on a thick smear bearing phagocytosed hemozoin; log transformed data.

[c] data are missing for 16 clients.

[d] analysis omits six patients for whom histological analysis was unavailable.

[e] reported use of antipyretics is categorized as no antimalarial drug use.

Table S5. Univariate logistic regression analysis of sequence type dominance.

	Type 1		Type 2		Type 3		Type 4	
	**OR (CI)**	***P***	**OR (CI)**	***P***	**OR (CI)**	***P***	**OR (CI)**	***P***
**Continuous variables**								
Age	1.09 (0.951, 1.24)	0.224	1.00 (0.861, 1.17)	0.964	*1.17 (0.989, 1.26)*	*0.0752*	**0.867 (0.766, 0.982)**	**0.0245**
Gravidity	*1.43 (0.965, 2.11)*	*0.0745*	0.538 (0.236, 1.23)	0.140	1.34 (0.941, 1.91)	0.104	*0.746 (0.528, 1.05)*	*0.0949*
Placental parasite density [a]	1.08 (0.652, 1.78)	0.775	1.46 (0.740, 2.90)	0.274	**0.585 (0.382, 0.895)**	**0.0136**	1.23 (0.870, 1.74)	0.240
Percent placental parasitemia [a]	1.00 (0.558, 1.80)	0.998	1.53 (0.741, 3.14)	0.252	**0.556 (0.338, 0.916)**	**0.0211**	1.27 (0.855, 1.89)	0.235
Placental hemozoin burden [b]	0.829 (0.404, 1.70)	0.610	2.46 (0.680, 8.93)	0.170	**0.548 (0.301, 0.997)**	**0.0490**	1.31 (0.780, 2.20)	0.308
Peripheral hemoglobin [c]	0.716 (0.374, 1.37)	0.314	0.995 (0.635, 1.56)	0.983	0.990 (0.680, 1.44)	0.959	1.10 (0.792, 1.52)	0.581
Infant birth weight	1.00 (0.998, 1.00)	0.881	**0.998 (0.995, 1.00)**	**0.0403**	1.00 (0.999, 1.00)	0.273	1.00 (0.999, 1.00)	0.506
Gestational age at birth	**0.431 (0.202, 0.922)**	**0.0300**	0.951 (0.478, 1.89)	0.885	1.16 (0.636, 2.11)	0.630	1.48 (0.879, 2.47)	0.141
**Categorical variables**								
Gravidity group	*18.1 (0.912, 358)*	*0.0575*	*0.114 (0.015, 1.34)*	*0.0886*	*4.67 (0.876, 24.8)*	*0.0711*	**0.273 (0.083, 0.898)**	**0.0325**
Upper quartile parasite density	0.206 (0.009, 4.50)	0.316	2.19 (0.339, 14.1)	0.410	0.134 (0.006, 2.85)	0.198	*4.67 (0.876, 24.9)*	*0.0710*
Upper quartile percent parasitemia	0.312 (0.013, 7.23)	0.468	1.00 (0.101, 9.93)	1.00	0.171 (0.008, 3.82)	0.265	*7.74 (0.870, 68.8)*	*0.0665*
Upper quartile hemozoin-WBCs	0.510 (0.019, 13.6)	0.688	*6.34 (0.804, 49.9)*	*0.0797*	0.917 (0.091, 9.25)	0.941	0.551 (0.083, 3.64)	0.536
Placental histology group [d]	0.542 (0.095, 3.08)	0.489	6.32 (0.291, 137)	0.240	*0.290 (0.067, 1.26)*	*0.0980*	1.87 (0.535, 6.53)	0.327
Anemia	1.29 (0.074, 22.4)	0.863	2.13 (0.307, 14.7)	0.445	1.36 (0.278, 6.68)	0.702	0.510 (0.129, 2.02)	0.339
HIV serostatus	2.96 (0.448, 19.5)	0.260	0.374 (0.016, 8.96)	0.544	0.611 (0.065, 5.75)	0.667	1.21 (0.241, 6.09)	0.815
Self-reported antimalarial drug use [e]	**0.182 (0.034, 0.980)**	**0.0473**	0.582 (0.101, 3.34)	0.544	3.67 (0.414, 32.5)	0.243	1.75 (0.466, 6.57)	0.407

Low birthweight	0.571 (0.020, 16.4)	0.744	**42.0 (3.29, 537)**	**0.0040**	0.376 (0.013, 10.6)	0.566	0.267 (0.026, 2.76)	0.268
Preterm birth	**15.6 (1.19, 205)**	**0.0365**	3.90 (0.297, 51.2)	0.300	0.470 (0.014, 15.4)	0.671	0.120 (0.004, 3.85)	0.231

Univariate regression analysis with group membership as dependent variable and continuous (linear regression) and categorical (logistic regression) variables as predictors. Statistically significant results are bolded; trending results are italicized.

OR (CI) = Odds Ratio (95% confidence interval). NA = not applicable. Results with p <0.05 are bolded and results with $0.05 < p < 0.1$ are italicized. All results with $p < 0.1$ are presented graphically in Figure 4A.

Among categorical variables gravidity group tests multigravid status, parasitemia and hemozoin analyses test presence in the upper quartile, histology group tests chronic PM, anemia tests hemoglobin < 11 g/dL, HIV tests HIV seropositivity, self-reported antimalarial drug use tests reported use of drugs, low birth weight tests birth weight ≤2500 g, and preterm birth tests gestational age <37 weeks.

[a] determined as summarized in Table 1 and Methods; parasitemia analyses done using log-transformed data. Percent placental parasitemia analysis omits one multigravida for whom a placental thin smear was unavailable.

[b] percent of white blood cells on a thick smear bearing phagocytosed hemozoin; log transformed data.

[c] data are missing for 16 clients.

[d] analysis omits six patients for whom histological analysis was unavailable.

[e] reported use of antipyretics is categorized as no antimalarial drug use.

Table S6. Multiple logistic regression analysis of sequence type dominance.

	Type 1		Type 2		Type 3		Type 4	
	**OR (CI)**	***P***	**OR (CI)**	***P***	**OR (CI)**	***P***	**OR (CI)**	***P***
**Continuous variables**								
Age	NA		NA	-	1.07 (0.885, 1.30)	0.477	**0.784 (0.616, 0.998)**	**0.0486**
Gravidity	NA	-	NA	-	NA	-	1.53 (0.771, 3.02)	0.225
Placental parasite density [a]	NA	-	NA	-	**0.139 (0.021, 0.918)**	**0.0405**	NA	-
Placental hemozoin burden [b]	NA	-	NA	-	*9.51 (0.701, 129)*	*0.0904*	NA	-
Gestational age at birth	0.537 (0.254, 1.14)	0.104	NA	-	NA	-	NA	-
**Categorical variables**								
Gravidity group	*23.6 (0.91, 610)*	*0.0570*	0.22 (0.014, 3.48)	0.283	0.816 (0.071, 9.33)	0.870	NA	-
Upper quartile percent parasitemia	NA	-	NA	-	NA	-	11.8 (0.594, 233)	0.106
Upper quartile hemozoin–WBCs	NA	-	10.4 (0.601, 179)	0.107	NA	-	NA	-
Placental histology group [c]	NA	-	NA	-	0.399 (0.04, 4.00)	0.435	NA	-
Self-reported antimalarial drug use [d]	**0.128 (0.017, 0.992)**	**0.0491**	NA	-	NA	-	NA	-
Low birthweight	NA	-	**22.1 (1.31, 373)**	**0.0319**	NA	-	NA	-

Multiple regression analysis with group membership as dependent variable and continuous (linear regression) and categorical (logistic regression) variables as predictors. Statistically significant results are bolded; trending results are italicized.

OR (CI) = Odds Ratio (95% confidence interval). NA = not applicable. Results with p <0.05 are bolded and results with 0.05 < p < 0.1 are italicized. Analyses considered variables with p < 0.1 in univariate analysis, and all outcomes are shown in Figure 4B.

[a] determined as summarized in Table 1 and Methods; parasitemia analyses done using log-transformed data.

[b] percent of white blood cells on a thick smear bearing phagocytosed hemozoin; log transformed data.

[c] analysis omits six patients for whom histological analysis was unavailable.

[d] reported use of antipyretics is categorized as no antimalarial drug use.

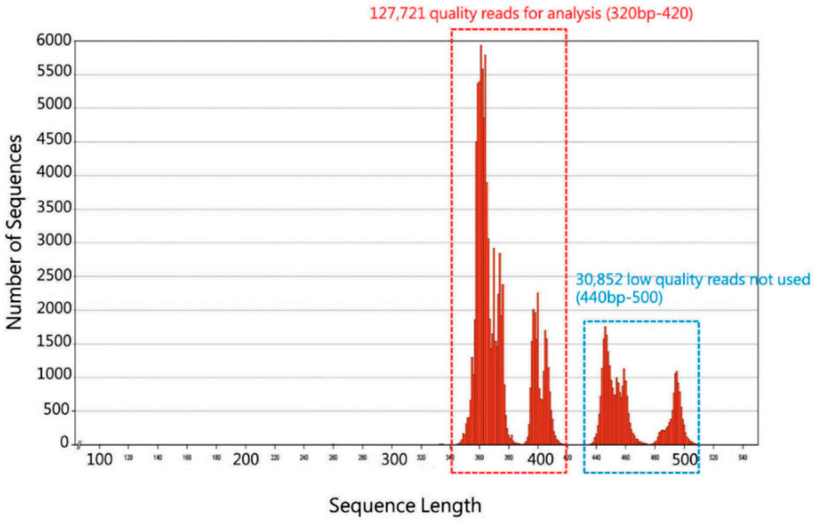

Figure S1. Summary of total number of quality reads used. Shown on the x-axis is the total number of sequences and y-axis the sequence lengths in base pairs (bp).

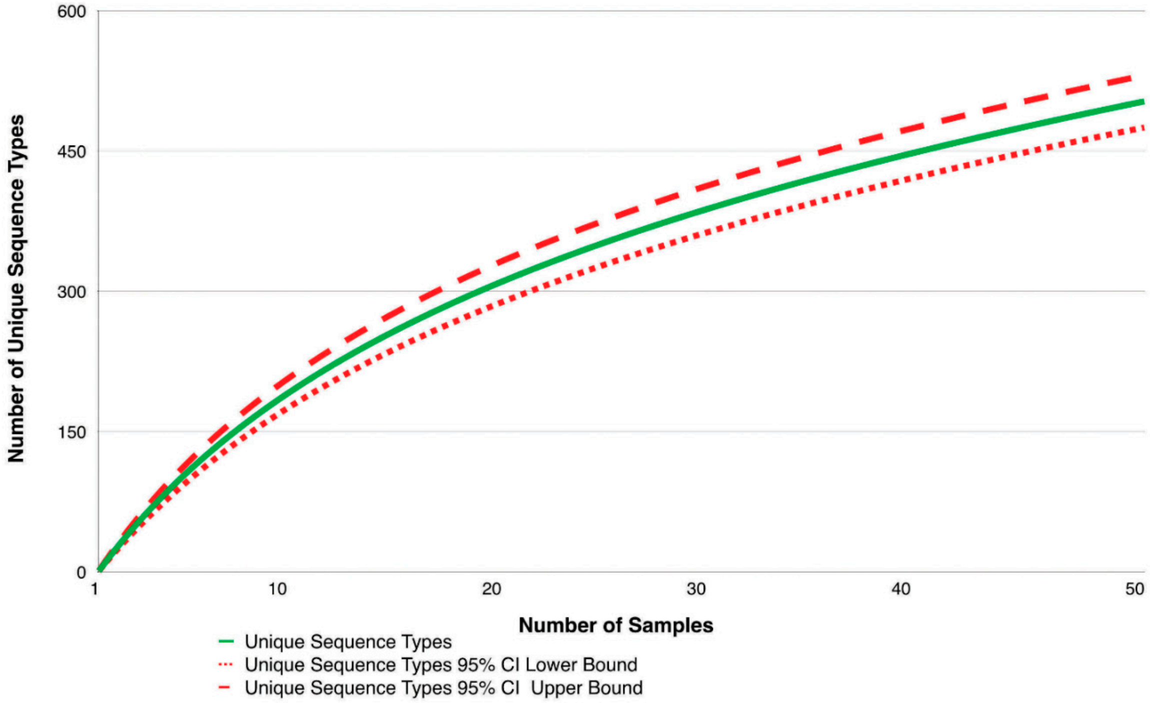

Figure S2. Rarefaction curve of *var2csa* unique sequence types for entire study population (N = 49). The computed refraction curve (in solid green) shows the expected average rate of unique sequence types that would be produced as a result of repeated deep sequencing. Upper and lower 95% confidence intervals (CI) for species richness are shown by dashed and dotted red lines, respectively.

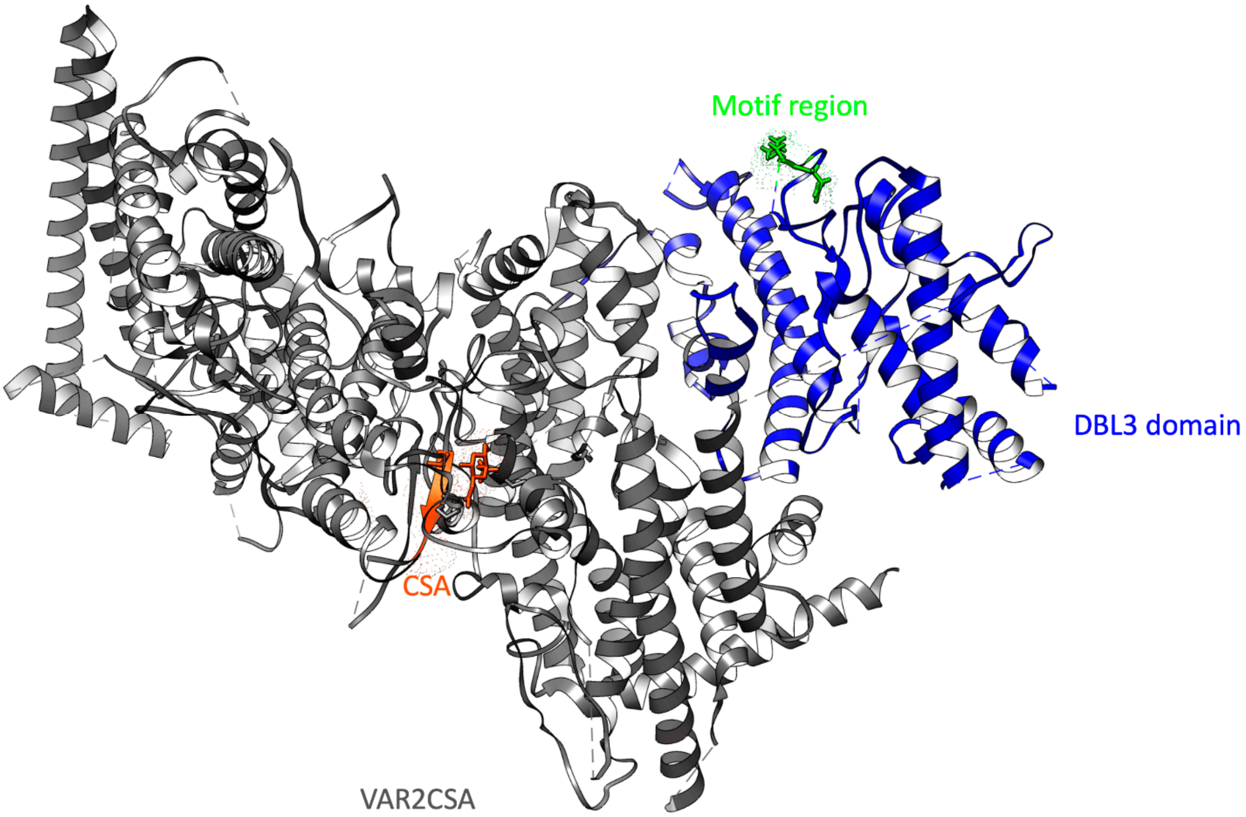

Figure S3. VAR2CSA and DBL3X motif region. A ribbon representation of VAR2CSA (grey), DBL3X domain (blue). Dotted circles and atom stick representation show the CSA binding site (orange) and specific motif region (green) associated with gravidity and placental malaria outcomes.

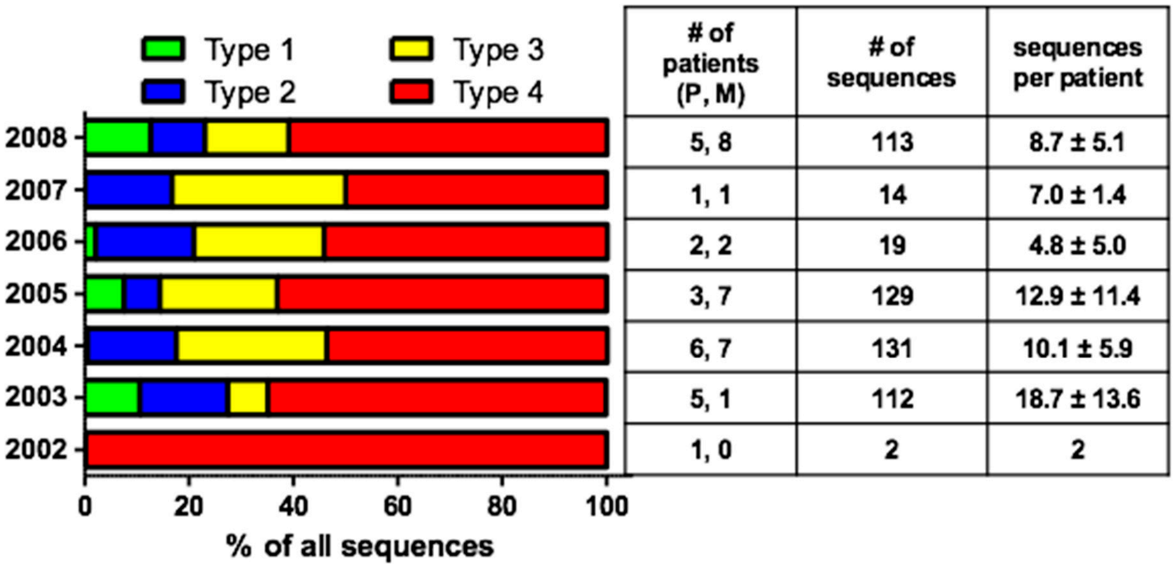

# of patients (P, M)	# of sequences	sequences per patient
5, 8	113	8.7 ± 5.1
1, 1	14	7.0 ± 1.4
2, 2	19	4.8 ± 5.0
3, 7	129	12.9 ± 11.4
6, 7	131	10.1 ± 5.9
5, 1	112	18.7 ± 13.6
1, 0	2	2

Figure S4. DBL3X sequence types are consistently found independently of time and location of patient recruitment. The graph depicts proportional distribution of sequence types by year of patient recruitment. The tale at right summarizes number of patients recruited each year (stratified by gravidity group), number of unique sequences contributed by those patients, and the mean number of unique sequences per patient.

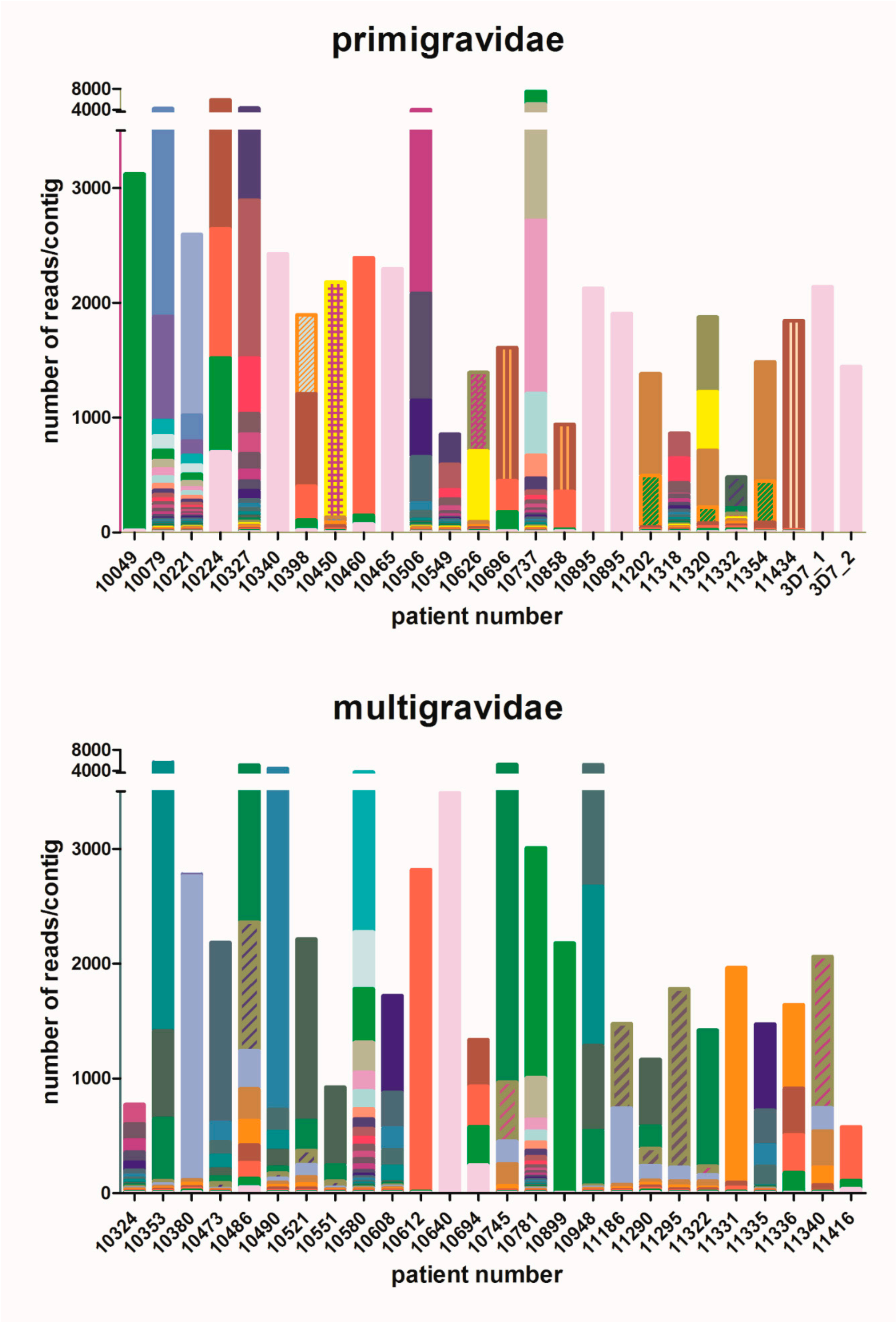

Figure S5. Unique DBL3X sequences are not proportionally represented at the individual patient level. The graphs depict the number of unique contigs within each patient. Notably, most patients have a single dominant contig. Patient 895 is depicted twice, representing two separate deep sequencing runs. This patient was previously identified (REF) as having a single unique sequence. DNA from the clonal laboratory isolate, 3D7, was also included as a control, and shows, in two

separate runs, a single contig. Similar colors across individual patients do *not* imply sequence similarity.

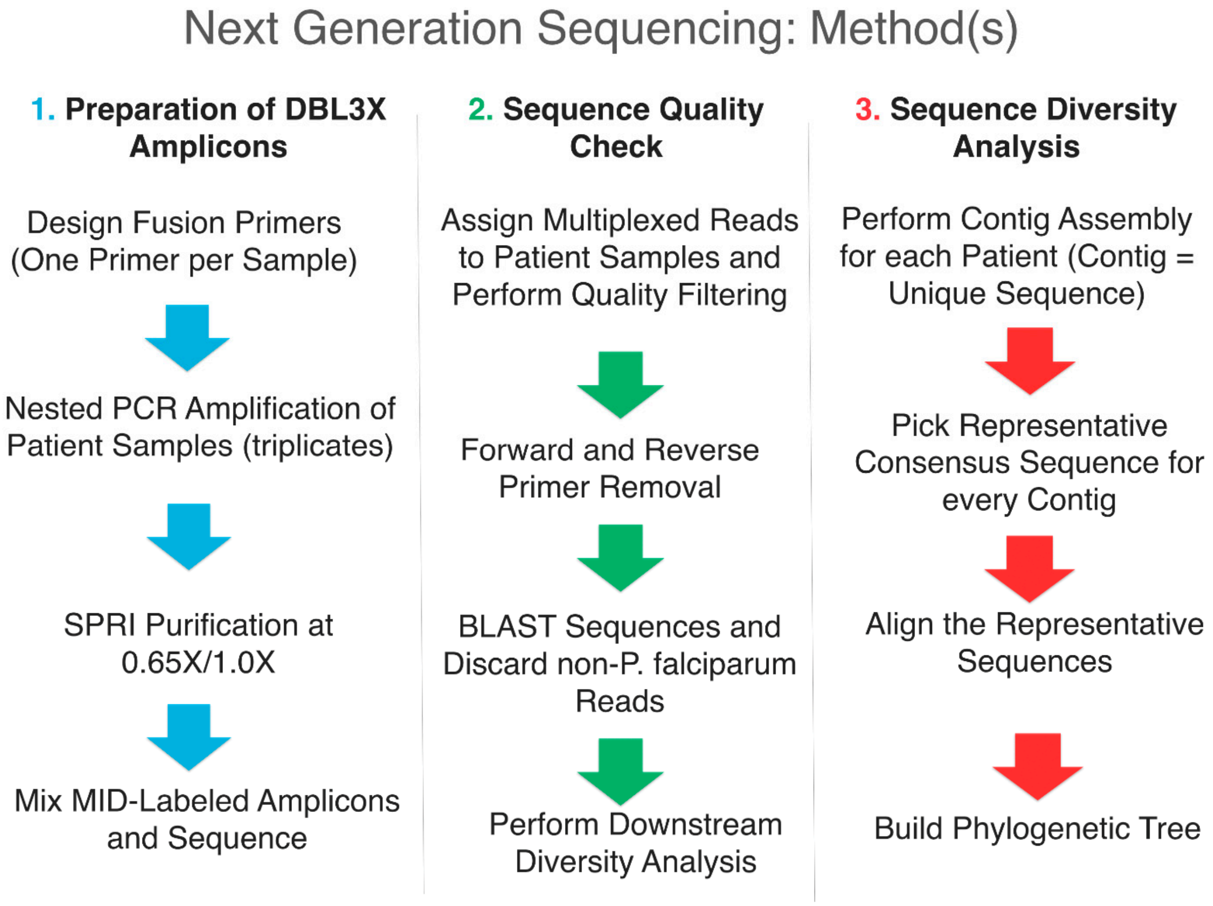

Figure S6. Schematic overview of the overall sequencing process.
